# Research on Multi-Dimensional Bionic Design of Flexible ECG Electrodes for Wearable Monitoring

**DOI:** 10.3390/s26154998

**Published:** 2026-08-06

**Authors:** Changpo Zhang, Zeyu Liu, Zheng Zhao, Feng Chen, Chengcheng Liu, Jin Yu

**Affiliations:** 1 School of Mechanical Engineering, University of Jinan, Jinan 250022, China; 2 Shandong Key Laboratory of Surface Engineering and Intelligent Equipment for Key Metal Components, University of Jinan, Jinan 250022, China

**Keywords:** flexible ECG electrodes, biomimetic design, wearable monitoring, electrode–skin interface, ECG monitoring

## Abstract

With the rapid advancement of wearable medical technologies, long-term, dynamic, non-invasive high-precision electrocardiogram (ECG) monitoring has emerged as a critical requirement across clinical and civilian healthcare domains in both clinical and civilian applications. However, conventional Ag/AgCl wet electrodes and common flexible dry electrodes suffer from critical limitations, including high electrode–skin interfacial impedance, severe motion artifacts, inadequate biocompatibility, and poor long-term wearing comfort, which severely restrict their practical performance. The performance improvement of ECG electrodes relies on the synergistic realization of low interfacial impedance, strong anti-interference capability, excellent biocompatibility, and satisfactory long-term wearability. Biomimetic design provides a pivotal strategy for addressing the incompatibilities at the electrode–skin interface and breaking through the performance bottlenecks of traditional electrodes. This paper systematically reviews the fundamental theories and key design principles of biomimetic ECG electrodes, and elaborates the biomimetic prototypes, design mechanisms, and typical application cases from five core perspectives: structural biomimetics, interfacial biomimetics, mechanical biomimetics, functional biomimetics, and material biomimetics. Furthermore, the application progress of biomimetic electrodes in civil wearable devices, clinical monitoring, and special scenarios is summarized, together with an analysis of the current industrialization layout and patent status. Finally, future development trends are prospected, including multi-dimensional synergistic biomimetics, intelligent biomimetics, green biomimetics, clinical-grade biomimetics, and low-cost scalable fabrication. This review supplies systematic theoretical underpinnings and practical guidance for the design optimization, performance enhancement, and industrial application of biomimetic ECG electrodes, and is of great significance for promoting the evolution of ECG monitoring toward high precision, long-term operation, and convenient utilization.

## 1. Introduction

As a core physiological indicator reflecting the electrical activity of the heart and assisting in the diagnosis of cardiovascular diseases and assessment of health status, ECG signals play an irreplaceable role in clinical diagnosis and treatment as well as daily health management [1]. With the continuous development of medical and health undertakings and the gradual improvement of public health awareness, the application scenarios of ECG monitoring are no longer limited to precise monitoring in clinical settings of hospitals, but have gradually extended to the field of civil daily screening, forming a diversified and comprehensive monitoring demand system. Currently, wearable, long-term, dynamic, and non-invasive have become the core demand orientations of ECG monitoring. Whether it is the continuous monitoring of critically ill patients and post-operative rehabilitation populations in clinical scenarios, or the daily health monitoring of the general population in civil scenarios, higher requirements are put forward for the convenience, comfort, and continuity of ECG monitoring equipment, which further highlights the importance of ECG electrodes as the core component for signal acquisition [2].

Traditional ECG electrodes widely used in current clinical and civil fields still have many significant limitations, making it difficult to fit the criteria for prolonged cutaneous attachment. Among them, Ag/AgCl wet electrodes, as the most widely used electrode type in clinical applications, have relatively stable signal acquisition performance but rely on conductive gel to achieve effective contact with the skin. However, the conductive gel is prone to drying and falling off during long-term wearing, which not only reduces the contact stability between the electrode and the skin, affects the continuity and accuracy of signal acquisition, but may also irritate the skin and cause allergic reactions, failing to fit the criteria for prolonged cutaneous attachment for long-term continuous wearing. Signal fluctuations are also likely to occur in dynamic monitoring scenarios [3]. Although ordinary flexible dry electrodes do not rely on conductive gel, their wearing convenience is greatly improved, and they can better adapt to the application needs of wearable devices. However, limited by their own structural and material characteristics, their contact effect with the skin interface is poor, with high interfacial impedance and weak anti-interference ability. During human activities, they are easily affected by factors such as friction and sweat, leading to problems such as clutter and distortion of ECG signals, which also do not fully fit the criteria for prolonged cutaneous attachment and long-term dynamic monitoring [4]. The design of high-performance ECG electrodes needs to take into account various core requirements: low interfacial impedance is the basis for ensuring efficient and accurate acquisition of ECG signals, which can effectively reduce signal attenuation and ensure signal integrity; strong anti-interference ability can resist various interference factors such as external electromagnetic environment, human motion friction, and electrical activity on the skin surface, ensuring signal stability during dynamic monitoring; good biocompatibility can avoid adverse reactions such as irritation and allergies when the electrode is in long-term contact with the skin, adapting to the needs of people with different skin types; and long-term wear ability requires the electrode to have the characteristics of light weight, flexible fit, breathability, and durability, which can adapt to various changes in human movement and posture and realize long-term continuous and stable signal acquisition [5].

The inherent flaws of conventional electrodes fundamentally arise from inadequate adaptability at the electrode–skin interface: electrode architectures and constituent materials fail to conform to skin microtopography or accommodate dynamic cutaneous deformation, which in turn triggers a suite of issues including poor conformal contact and distorted electrophysiological waveforms [6]. To this end, the concept of biomimetic design has been introduced into the research and development of ECG electrodes. This concept takes the microstructure, physiological characteristics, and mechanical properties of human skin as the imitation objects, and optimizes the structural design and material selection of the electrode [7]. By simulating the flexibility, skin-friendliness, and breathability of the skin, it further improves the interface contact state between the electrode and the skin, optimizes the interface action mechanism, fundamentally solves the adaptability problem of the electrode–skin interface, effectively improves the comprehensive performance of ECG electrodes, and becomes a key idea for breaking through the limitations of traditional electrodes and improving the quality of ECG monitoring. It also provides a new technical path for the development of high-performance ECG electrodes, helping to promote the development of wearable ECG monitoring equipment towards a more accurate, comfortable, and practical direction, and better fit the criteria for prolonged cutaneous attachment in clinical and civil fields [2].

This review first combs through the development history of biomimetic ECG electrodes and systematically evaluates the core performance indicators of such electrodes. Subsequently, combined with specific research examples, the core design dimensions of biomimetic ECG electrodes are elaborated in detail; finally, their application scenarios are introduced through typical cases, and the latest research progress and future development prospects in the field of biomimetic ECG electrodes are discussed, emphasizing the innovation and improvement potential of this emerging field. All subsequent analyses on mechanisms, structures, and applications revolve around body-surface ECG electrodes. Implantable devices, electromyography (EMG) sensors, and multimodal electronic skins are merely analogous cases for bionic design.

## 2. Development

### 2.1. Recent Developments

In recent years, with the rapid development of flexible electronics and wearable biosensing technologies, ECG signals—one of the most clinically significant and practically feasible physiological signals—have gained increasing importance in cardiovascular screening, sports monitoring, rehabilitation, and long-term health management. However, ECG reliability is highly dependent on the stability of the skin–electrode interface. Conventional Ag/AgCl gel electrodes, though effective in short-term static measurements, suffer from gel dehydration, skin irritation, sweat-induced adhesion failure, and severe motion artifacts under dynamic conditions, limiting their applicability in continuous wearable scenarios. To address these challenges, dry flexible ECG electrodes have emerged, among which biomimetic ECG electrodes have developed most rapidly. By mimicking natural strategies for adhesion, perspiration, and adaptive regulation, these electrodes aim to construct skin-compatible interfaces that simultaneously improve impedance, comfort, adhesion, motion tolerance, and durability.

**Figure 1:**
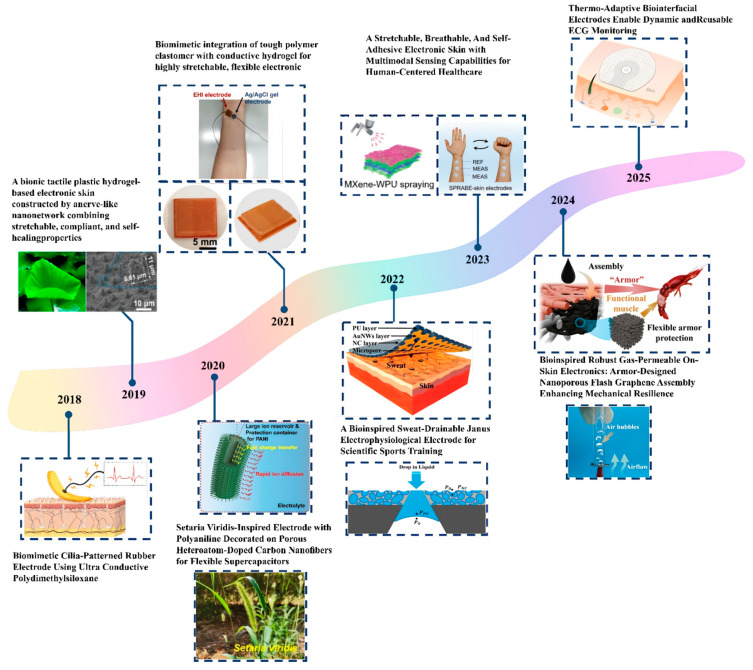
The evolution of ECG electrode simulation. Image for “the evolution of ECG electrode simulation” [8]. Copyright 2018 Adv. Funct. Mater. Image for “flexible capacitive tactile sensor based on the micropatterned flexible PDMS substrate duplicated from the lotus leaves” [9]. Copyright 2019 Acc. Chem. Res. Image for “illustration of PANI-decorated porous hybrid electrodes mimicked by the *S. Veridic* structure” [10]. Image for “seteria veridic-inspired electrode with polyaniline decorated on porous heteroatom-doped carbon nanofibers for flexible supercapacitors” [10]. Copyright 2020 ACS Applied Materials & Interfaces. Image for “optical photograph of the EHI with square length of 1 cm”. Image for “schematic illustration of EHI electrode and Ag/AgCl gel electrode for ECG detection” [11]. Copyright 2021 Nano Energy. Image for “schematics depicting the sweat accumulated or absorbed at the skin–electrode interface covered with a conical pore electrode, respectively”. Image for “principle schematic of directional water transport through the conical pores”. Copyright 2022 Advanced Materials Technologies. Image for “fabrication process of the SPRABE-skin based on electrospinning and spraying (**top**). Note that the TPU membranes were electrospun onto both sides of the S-layer to serve as P-layer and I-layer” [12]. Image for “SPRABE-skin electrodes attached at wrist flexor muscles for EMG measurement using bipolar signal acquisition configuration [12]”. Copyright 2023 Advanced Functional Materials. Image for ‘armor’ design strategy for the FG/PPMF electrode, where PPMF acts as a protective layer for the internal FG components, forming a porous conductive network. This resembles a crayfish shell protecting the crayfish muscle. The turbotrain characteristics of FG facilitate the formation of this porous structure. FG, in the form of an ink, is assembled in situ deep within the PPMF to create FG/PPMF [13]”. Image for “photograph demonstrates the gas permeability of FG/PPMF [13].” Copyright 2024 Advanced Science. Image for “structure, preparation and application of TAB electrode [14]”. Copyright 2025 ACS Applied Materials & Interfaces. Refer to the complete evolution context of biomimetic electrodes from 2018 to 2025; mature bionic technologies can be selected for ECG electrodes according to scenarios. Skin microstructures are adopted for short-term monitoring to expand contact area and cut impedance; Janus unidirectional sweat transport structures are applied for long-term monitoring to avoid baseline drift from sweat accumulation; shrimp shell armor porous structures fit exercise monitoring to balance air permeability and conductive network protection; thermo-adaptive interfaces are suitable for reusable electrodes. The core inspiration is that single-structure optimization is insufficient, and coordinated design of materials, structures, and functions is required.

From 2018 to 2025, the evolution of biomimetic ECG electrodes presents a clear chronological trajectory driven by successive practical pain points, with each technical stage strictly corresponding to the nodes in Figure 1. In 2018, to address the poor conformal contact between planar electrodes and rough skin, researchers developed biomimetic cilia-patterned ultra-conductive polydimethylsiloxane (PDMS) electrodes (2018 node, Figure 1). The microcilia structure enlarged the effective contact area and reduced interfacial impedance, laying the structural basis for stable ECG acquisition [8]. In 2019, targeting the mechanical mismatch between rigid electrode materials and soft skin tissue, a bionic hydrogel electronic skin with a nerve-like conductive nanonetwork was proposed (2019 node, Figure 1). It achieved mechanical matching with skin, integrating stretchability, compliance, and self-healing properties to maintain stable signal transmission under deformation [9]. In 2020, driven by the demand for long-term cyclic service, a *Setaria viridis*—inspired porous carbon nanofiber electrode—was designed (2020 node, Figure 1). The bionic porous architecture enhanced mechanical resilience and conductive stability, and also supported the integration of flexible energy storage units [10]. In 2021, to balance mechanical support and electrical performance in dynamic scenarios, a skin-inspired layered structure combining a tough polymer elastomer and conductive hydrogel was constructed (2021 node, Figure 1), realizing synergistic improvement of mechanical robustness and dynamic signal stability [11]. In 2022, focusing on sweat-induced signal distortion and adhesion failure during exercise, a sweat-drainable Janus electrophysiological electrode was developed for sports training (2022 node, Figure 1). Its unidirectional sweat transport structure keeps the sensing interface dry and suppresses sweat-related motion artifacts [11]. In 2023, to fit the criteria for prolonged cutaneous attachment, a stretchable, breathable, and self-adhesive multimodal sensing electronic skin was proposed (2023 node, Figure 1). It integrates ECG monitoring with multi-physiological sensing and supports comfortable long-term wear [12]. In 2024, aiming at performance degradation of conductive networks under repeated mechanical stress, a bioinspired armor-structured nanoporous graphene on-skin electrode was fabricated (2024 node, Figure 1). It notably improves electrode durability and reusability while retaining gas permeability [13]. In 2025, to adapt to dynamically changing skin states in complex scenarios, thermo-adaptive biointerfacial electrodes emerged (2025 node, Figure 1). They dynamically regulate interfacial adhesion and sweat transport via thermal response, enabling stable dynamic ECG monitoring [14]. Overall, biomimetic ECG electrodes have evolved from single structural/material optimization to functional integration and adaptive interface design, systematically addressing core interfacial challenges. Future research will focus on multi-scale biomimetic strategies, adaptive feedback regulation, long-term biocompatibility, and deep integration with intelligent wearable systems.

### 2.2. Analysis of the Principle of Bionic Electrodes

The core working principle of PULSE flexible devices lies in the trinity of mechanical matching, mechanoelectrical coupling, and high-fidelity electrophysiological recording, designed for long-term mechanosensitive electrophysiological monitoring of cardiomyocytes and tissues. Its mechanical architecture adopts a bilayer structure: the bottom layer consists of soft PDMS gel with a modulus matching that of the myocardial extracellular matrix (ECM); the top layer is a 450 nm ultrathin SEBS/PDMS nanomembrane. Although the intrinsic modulus of the bulk material is relatively high, the nanoscale thickness tunes the overall device modulus to approximately 10 kPa, achieving perfect mechanical matching with myocardial tissue. This ultrathin interface contracts and relaxes synchronously with cardiomyocytes, eliminating mechanical interference and contraction constraints imposed by conventional rigid electrodes. Embedded gold microcircuits form interconnected non-parallel microcracks upon stretching, maintaining stable conductive pathways under large strains. After strain training, impedance remains stable within the physiological stretch range. At the cell–interface coupling level, cardiomyocytes sense substrate stiffness via integrin β1 and focal adhesion proteins. The soft PULSE substrate activates physiological mechanotransductive signaling, promoting ordered sarcomere assembly and high expression of the gap junction protein Cx43, accelerating cardiomyocyte maturation and establishing a positive feedback loop between contraction and electrical signals: unimpeded cellular contraction pulls the flexible substrate, enabling normal gating of ion channels in the cell membrane and significantly increasing field potential amplitude. In contrast, rigid substrates restrict contraction, impede ion transport, and produce weak, distorted electrical signals. For electrophysiological recording, the device captures myocardial field potentials using microelectrodes, with ultrathin encapsulation exposing only electrode sites to achieve seamless tissue–electrode contact and greatly reduce gap noise. Additionally, self-healing SEBS encapsulation and strong interfacial bonding with PDMS ensure long-term stability in aqueous environments, allowing continuous multiday recording without signal attenuation. The device exhibits high sensitivity to drug responses and arrhythmia models, faithfully recapitulating myocardial physiological and pathological phenotypes.

**Figure 2:**
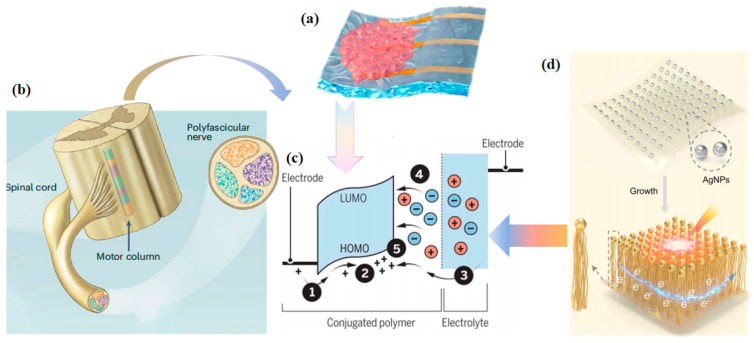
The principle of bionic electrodes. (**a**) The bilayer construct of PULSE combines a 450 nm-ultrathin nanofilm with a soft gel substrate, yielding exceptional stretchability, conductivity, and a modulus comparable to native heart tissue [15]. Copyright 2026 Science Advances. (**b**) Biointerface based on TMR of a polyfascicular nerve and the use of a microelectrode array for recording and decoding [16]. Copyright 2025 Nature Biomedical Engineering. (**c**) An energy-level diagram of a microelectrode operation in the electrolyte with five phases: (1) a hole is injected from the metal electrode into the HOMO level of the polymer (HOMO and LUMO refer to highest occupied and lowest unoccupied molecular orbitals, respectively); (2) the hole is transported in the delocalized HOMO level in the polymer; (3) the anion is driven into the polymer by the potential; (4) anion travel within the free volume between the polymer chains and crystals; and (5) electrostatic compensation of holes and anions at the combination site in the bulk [17]. Copyright 2022 Materials Science & Engineering R. (**d**) Schematic illustration of the seed-mediated growth strategy [18]. Copyright 2026 Science Advances.

TMR transfers multiple residual nerve fascicles following amputation to denervated host muscles (e.g., pectoralis minor, biceps brachii) (Figure 2b). Nerve axons regenerate and reinnervate the muscle, forming hyperinnervation such that multiple neural signals originally governing distinct movements—including finger flexion/extension, wrist rotation, and elbow motion—converge within the same muscle, which acts as a natural biological signal amplifier. For signal acquisition, a 40-channel high-density linear microelectrode array is implanted into the reinnervated muscle, achieving high spatiotemporal resolution intramuscular EMG recording capable of resolving single motor unit firings and overcoming the limited spatial resolution of surface electrodes. Three core mechanisms in this subsection can be transferred to surface ECG electrodes. First, the gradient ultrathin film scheme of PULSE matches the epidermal modulus to reduce interfacial shear artifacts during movement. Second, the bulk synergistic conduction mechanism of organic mixed ionic–electronic conductors (OMIEC) breaks the limitation of only relying on skin double-layer coupling, effectively lowering low-frequency impedance and capturing faint ECG waveforms. Third, the 3D conductive network of vertical Au-Ag nanowires maintains unbroken pathways under stretching and bending, greatly improving signal integrity in daily activities.

The core decoding pipeline consists of multiple layers: first, blind source separation (BSS) decomposes mixed EMG signals into individual motor unit (MU) spike trains, removing noise and interference; second, motor unit tracking distinguishes task-specific MUs from shared MUs based on action potential morphology and spatial distribution; third, cross-correlation analysis quantifies MU synchronization, enabling clustering into functional groups corresponding to descending central drive, demonstrating that distinct movements within the same muscle are independently encoded by different MU ensembles; and finally, non-negative matrix factorization (NNMF) reduces high-dimensional MU activity to a low-dimensional neural manifold, where each latent dimension corresponds to an independent motor command. This approach decouples multiple independent control signals from a single muscle without nerve transection or additional muscle grafts. These signals can be directly mapped to multi-degree-of-freedom prosthetic actions including finger extension/flexion, wrist flexion/extension, and pronation/supination, achieving highly controllable and robust neural prosthetic control.

Electrodes employing (Figure 2c) OMIECs—typified by PEDOT:PSS—as their core functional layer enable efficient cross-modal transduction between biological ionic signals and electronic signals, which distinguishes them fundamentally from conventional metal electrodes that rely solely on interfacial double-layer coupling. OMIECs possess the unique ability to conduct both ions and electrons within their bulk phase. Their conjugated polymer backbones provide continuous π-conjugated pathways for efficient transport of holes and electrons, enabling electronic conduction. Meanwhile, free volume between polymer chains and hydrophilic side chains permits the injection, diffusion, and migration of hydrated ions throughout the material bulk, rather than restricting ionic motion to the electrode surface alone. In a three-electrode configuration, the complete transduction process proceeds in five key steps: holes are first injected into the HOMO level of the OMIEC via the external circuit and rapidly delocalize along the conjugated backbone; driven by the applied potential, anions from the electrolyte migrate into the polymer film and diffuse through the interchain free volume; and finally, electrostatic charge compensation occurs between holes and anions within the bulk, completing the conversion from ionic to electronic signals. This process is characterized electrochemically by dominant volumetric capacitance, which is far higher than that of traditional metal electrodes, accompanied by significantly reduced interfacial impedance—especially exceptionally low-frequency impedance behavior matching the frequency range of bioelectrical signals. Cyclic voltammograms exhibit a typical rectangular box shape, indicative of a non-Faradaic capacitive process without redox charge transfer, thus ensuring high stability and minimal artifacts suitable for long-term extracellular electrophysiological recording. Furthermore, organic mixed ionic–electronic electrodes (OMEAs) possess Young’s moduli close to those of biological tissues and lack rigid interfacial oxide barriers, enabling low-trauma, high-fidelity ionic–electronic coupling with cells and tissues. This greatly enhances the pickup capability and signal-to-noise ratio for weak bioelectrical signals at the microvolt level.

Vertically aligned Au–Ag nanowires enable dual-modal sensing through two complementary mechanisms (Figure 2d). First, Flexors optical sensing: high-density, uniformly distributed electromagnetic hotspots form between adjacent vertical nanowires, drastically enhancing Raman scattering signals. The intrinsic hydrophobicity of the substrate enables direct analyte enrichment, further boosting detection sensitivity. Nanowires are firmly anchored at their roots; under stretching, only controllable wrinkles and microcracks form, preserving hotspot integrity. SERS signals remain nearly unchanged under large strains and repeated cycling. Second, bioelectrical signal sensing: nanowires form a continuous three-dimensional conductive network on a porous sponge substrate, achieving low-impedance, intimate contact with skin or tissues without conductive gels, enabling stable acquisition of surface or ex vivo electrophysiological signals such as ECG and EMG with efficient electron transport and high signal fidelity. The overall device features morphological adaptability, stretch durability, and strain insensitivity, realizing mechanically robust multimodal flexible sensing. The core mechanism of TMR combined with high-density microelectrode-based neural decoding relies on three integrated components: targeted muscle reinnervation (TMR) surgery to restructure signal sources, high-density electrode recording, and mathematical decoding via blind source separation and matrix decomposition. This framework ultimately extracts multiple independent motor intentions from a single muscle, enabling precise multi-degree-of-freedom control of prosthetic limbs.

### 2.3. Evaluation Index

#### 2.3.1. Biocompatibility

Biocompatibility is a fundamental prerequisite for the safe, long-term use of bionic electrodes. Essentially, it refers to the ability of electrode materials to interact with the body at the molecular, cellular and tissue levels without triggering excessive immune rejection or inflammatory responses, nor causing cytotoxicity, genotoxicity or carcinogenicity, whilst maintaining their own functional stability. Relevant studies have shown that flexible electrodes prepared from silk protein do not induce significant inflammatory cell infiltration or fibrotic encapsulation in local tissues following implantation in animal models; the natural structure of silk protein is highly compatible with biological tissues, demonstrating excellent biocompatibility [19]. Concurrently, the researchers co-cultured the regenerated sericin-based hydrogel electrodes with cells in vitro. The data showed that the hydrogel extract exhibited a high survival rate for mouse embryonic fibroblasts, meeting the basic requirements for non-cytotoxic medical materials. In contrast, traditional electrodes cause mechanical damage due to mechanical mismatch, and during long-term use, the materials release trace amounts of toxic substances, leading to a foreign body inflammatory response [20]. Bionic electrodes composed of natural polymeric materials (such as cellulose, chitosan, and sericin) effectively reduce the body’s recognition of and immune rejection of foreign bodies due to their low immunogenicity and degradability. This provides a favorable physiological environment for the electrodes and demonstrates high biocompatibility [21]. ECG electrodes can draw on the biomimetic design of natural polymer materials. Silk fibroin, chitosan, alginate, and other substrates are selected to prepare electrode surface layers. Such materials have low immunogenicity and hardly cause skin redness and inflammation after long-term attachment. Implantable ECG monitoring devices can adopt these materials to reduce in vivo fibrotic encapsulation, solving irritation problems arising from long-term wearing and implantation from the perspective of biosafety.

#### 2.3.2. Long-Term Stability

Long-term stability is a key criterion for assessing whether a biomimetic electrode can function continuously in a physiological environment; it primarily examines the electrode’s ability to maintain structural integrity and stable electrical performance over extended periods of use. Within the human body, there is constant exposure to bodily fluids, ion exchange, and mechanical disturbances caused by tissue movement. These factors gradually alter the internal structure and interfacial state of the electrode materials, thereby affecting signal acquisition and the efficacy of electrical stimulation. During their operational life, electrodes may exhibit phenomena such as swelling, aging, and interfacial delamination. Long-term in vivo experiments have revealed that electrodes with poorly designed structures experience significant performance degradation within a few weeks of implantation, making them unsuitable for chronic monitoring and therapeutic applications. To address this issue, researchers have widely adopted biomimetic gradient modulus and helical array structures in the design of biomimetic electrodes. This design mimics the gradual transition from hard to soft found in biological soft tissues, significantly reducing the mechanical stress generated during tissue movement. It prevents electrode fracture or delamination at the interface caused by fatigue, thereby ensuring long-term durability at the structural level [22]. To address the issue of oxidation-induced degradation in conductive polymers, bio-inspired electrodes incorporating bio-inspired antioxidant composite systems have now been developed. By mimicking the antioxidant defense mechanisms found in living organisms, these systems significantly enhance the chemical stability of the materials under prolonged electrochemical stimulation, thereby slowing down performance degradation and enabling the electrodes to function reliably over the long term in complex physiological environments [23]. ECG electrodes can refer to two optimization schemes: gradient modulus and biomimetic antioxidation. The gradient layered structure disperses interfacial stress caused by skin movement to prevent electrode delamination and fracture. The biomimetic antioxidant composite system slows down the electrochemical aging of conductive polymers. Stable impedance and intact ECG waveforms can be maintained for a long time even in complex sweat and body fluid environments, suitable for multi-day continuous monitoring.

#### 2.3.3. Interfacial Impedance

Interfacial impedance is a core metric for evaluating the electrical coupling efficiency at the electrode–skin interface, essentially reflecting the degree of energy loss during the transmission of bioelectrical signals from the ionic conductor (skin tissue) to the electronic conductor (electrode). High interfacial impedance leads to signal attenuation, waveform distortion, and low-frequency drift, making it one of the key bottlenecks limiting ECG signal quality. From a physical perspective, the electrode–skin interface can be equivalent to a complex electrochemical system comprising contact resistance, charge transfer resistance, and an electric double layer capacitance. Its magnitude is highly dependent on the interfacial contact state, the effective contact area, and the efficiency of ion–electron coupling. Therefore, the design of biomimetic electrodes typically revolves around “increasing the effective contact area” and “optimizing the interfacial transmission pathway.” In terms of biomimetic strategies, existing research primarily reduces interfacial impedance through the following three approaches: First, at the structural biomimicry level, by constructing micro/nanostructures or porous structures (such as sponge-like or skin-texture biomimetic structures), the actual contact area between the electrode and skin is significantly increased, interfacial gaps are reduced, thereby lowering contact resistance and shortening the signal transmission path. Second, at the material and interfacial biomimicry level, conductive hydrogels or ionic conductive materials introduce ionic conduction pathways by simulating the high-water-content environment of biological tissues, achieving efficient coupling with the skin’s natural ionic system. This “ion–electron synergistic conduction” mechanism can effectively reduce interfacial charge transfer resistance and enhance the stability of low-frequency signal transmission. Furthermore, at the functional biomimicry level, such as in neural-like conductive network electrodes, a highly interconnected conductive network is constructed to maintain continuous conduction paths during deformation, thereby avoiding sudden increases in equivalent interfacial impedance caused by local disconnections. Layered structured electrodes, on the other hand, reduce interfacial micro-slippage through mechanical division of labor, suppressing impedance fluctuations at the system level. Breathable electrodes regulate the interfacial microenvironment to prevent impedance drift over time caused by sweat accumulation. The core mechanism is that interfacial impedance is determined not only by the conductivity of the material, but more fundamentally by the synergistic effect of “contact area—interfacial state—continuity of the transmission path.” By simultaneously optimizing these three dimensions, biomimetic design converts the unregulated contact and unstable electrode–skin interface observed in conventional electrodes into a system featuring well-defined contact and robust ionic–electronic coupling interfaces, thereby achieving a unification of low impedance and high stability. ECG electrodes can integrate three types of biomimetic paths to reduce impedance. Micro-nano porous morphology is constructed structurally to expand contact area. High-water ionic biomimetic layers are adopted materially to build ion–electron synergistic conduction channels. Interconnected conductive networks are designed functionally to keep pathways unbroken under deformation. The three methods jointly weaken signal attenuation and waveform distortion and improve the collection efficiency of faint ECG signals.

#### 2.3.4. Signal-to-Noise Ratio

Signal-to-noise ratio (SNR) is one of the core metrics for evaluating ECG signal quality, defined as the ratio of the amplitude of the effective ECG signal to the amplitude of background noise. A higher SNR indicates clearer P-QRS-T waveforms, enabling more reliable feature extraction and clinical interpretation. In practical applications, ECG noise primarily originates from the following sources: (i) low-frequency drift caused by unstable interfacial impedance; (ii) abrupt resistance change noise due to fractures in the conductive network; (iii) interfacial disturbances induced by motion; and (iv) changes in the interfacial state caused by sweat and environmental factors. Therefore, SNR is not a single electrical parameter but a comprehensive reflection of multiple coupled interfacial and structural factors. In biomimetic design, strategies for improving SNR are primarily manifested in the following aspects: First, in terms of conductive network biomimicry, neural-like conductive networks, by constructing highly interconnected and reconfigurable conductive pathways, maintain electrical continuity during stretching or bending, thereby avoiding instantaneous noise peaks caused by microcracks or local disconnections. This topological stability directly suppresses the generation of high-frequency noise. Second, at the interfacial biomimicry level, conductive hydrogels form a stable ion–electron coupling interface with the skin, which can reduce interfacial impedance and minimize signal attenuation, thereby increasing the amplitude of the effective signal and enhancing the overall SNR. Third, at the structural biomimicry level, porous or sponge-like structures not only increase the contact area but can also store electrolyte internally, maintaining a moist interfacial state and delaying performance degradation, thus sustaining a high SNR during long-term monitoring. Furthermore, at the microenvironmental biomimicry level, breathable electrodes, by regulating moisture vapor exchange, prevent sweat accumulation and stratum corneum hydration swelling, fundamentally suppressing the increase in low-frequency noise caused by changes in the interfacial state. The core mechanism can be summarized as follows: The improvement of SNR essentially stems from the dual regulation of “signal enhancement and noise suppression.” Biomimetic electrodes, on one hand, enhance effective signal transmission by reducing interfacial impedance, and on the other hand, suppress noise sources through structural stability and interfacial stability, thereby achieving a systematic improvement in overall signal quality. Beyond the electrode–skin interface, the robustness of the full signal acquisition chain—including embedded acquisition architecture and front-end circuit design—is another critical determinant of final signal integrity that cannot be overlooked. Recent studies on the design and simulation-based validation of embedded acquisition architectures for in situ PCB integrity monitoring in biomedical devices have demonstrated that signal quality depends not only on interfacial impedance optimization at the material level, but also on the electrical robustness of the entire measurement system.

Specifically, impedance matching between the biomimetic electrode and the acquisition circuit, the rational design of pre-amplification and filtering networks, the integrity of PCB trace routing, and electromagnetic interference immunity all directly shape the output SNR and baseline stability. Even with a high-performance biomimetic electrode interface, unreasonable embedded architecture design and insufficient PCB signal integrity will introduce additional circuit noise, inter-channel crosstalk, and signal attenuation, offsetting the performance gains brought by interfacial biomimetic optimization. For wearable dynamic monitoring scenarios, synergistic optimization of the biomimetic electrode interface and the embedded acquisition electronic system is a necessary condition to fully exploit the low-impedance and anti-artifact advantages of bionic electrodes, and to achieve stable, high-fidelity ECG signal output.

Multi-dimensional biomimetic noise reduction logic can be referenced in ECG electrode design. Neural-network conductive structures suppress instantaneous clutter caused by deformation. Ionic biomimetic interfaces cut interfacial impedance and boost the amplitude of effective signals. Porous breathable structures avoid baseline drift induced by sweat accumulation. Multiple methods simultaneously improve the definition of P-QRS-T waveforms and reduce errors in subsequent ECG interpretation.

#### 2.3.5. Motion Artifact Suppression

Motion artifacts are one of the primary sources of interference in wearable ECG monitoring. Their essence lies in micro-slippage, changes in contact area, and localized strain concentration at the electrode–skin interface under dynamic conditions, which lead to contact impedance fluctuations and result in low-frequency drift and signal distortion. Unlike interfacial impedance and SNR, motion artifacts place greater emphasis on dynamic stability—that is, the electrode’s ability to maintain stable signal output under real human motions (bending, stretching, twisting, friction, etc.). In biomimetic design, motion artifact suppression is primarily achieved through the following three approaches: First, at the mechanical biomimicry level, layered structured electrodes (elastomer–hydrogel systems) mimic the mechanical division of labor between the skin’s epidermis and dermis, transforming complex deformations into controlled strains through structural layering. The elastomer layer bears the primary stress, while the hydrogel layer maintains interfacial contact, thereby significantly reducing interfacial shear and slippage. This is currently one of the most direct and effective strategies for suppressing motion artifacts. Second, at the structural biomimicry level, micro/nanostructures (such as gecko setae, micropillar arrays, etc.) enhance interfacial anti-slip capability by increasing friction and the number of contact points. This allows the electrode to maintain stable adhesion under dynamic conditions, thereby reducing signal fluctuations caused by changes in the contact state. Third, at the material and conductive network biomimicry level, neural-like conductive networks maintain the continuity of conductive pathways during deformation through a “sliding-rearrangement-recontact” mechanism, suppressing noise caused by fractures from within the material. Meanwhile, the compliance of conductive hydrogels buffers microscopic skin irregularities and reduces localized strain concentration. Furthermore, at the microenvironmental biomimicry level, breathable electrodes, by wicking away sweat and dissipating heat, prevent sweat from forming a lubricating layer that could lead to interfacial slippage, thereby indirectly suppressing motion artifacts. The core mechanism can be summarized as follows: The fundamental source of motion artifacts is “interfacial instability.” Biomimetic design, through the three mechanisms of mechanical division of labor, structural anchoring, and material buffering, transforms the interface from being “slippage-dominated” to “deformation-dominated,” thereby achieving stable signal output. It is worth noting that motion artifact suppression is not only determined by the mechanical stability of the electrode–skin interface, but also affected by the anti-interference capability of the back-end acquisition electronics. A robust embedded acquisition architecture with stable power supply, reasonable shielding design, and in situ PCB integrity monitoring can further suppress system-level noise and signal drift caused by dynamic deformation, forming a two-pronged guarantee with the biomimetic interface design.

To intuitively compare the comprehensive performance of various ECG electrodes, quantitative comparison tables are established from two dimensions: electrical performance and biomechanical performance. Table 1 summarizes three key electrical indicators including interfacial impedance, signal-to-noise ratio, and motion artifact suppression. Table 2 compares biological and mechanical indicators such as biocompatibility, durability, and Young’s modulus. Traditional Ag/AgCl wet electrodes and conventional flexible dry electrodes exhibit obvious performance limitations. Wet electrodes can only collect signals stably for a short time, and their performance deteriorates sharply once the conductive gel dries. Conventional dry electrodes suffer high interfacial impedance, severe noise, and prominent baseline drift during dynamic measurement. Bionic electrodes designed based on a single dimension can improve partial performance targets in a targeted manner, yet trade-offs between different properties remain inevitable. By synergistically optimizing multi-mechanisms covering structure, interface, material, and mechanics, functional bionic ECG electrodes achieve low interfacial impedance, high signal-to-noise ratio, and outstanding motion artifact suppression, together with favorable biocompatibility, long-term durability, and skin-matched modulus. With the best overall performance, they represent a highly promising technical solution for long-term wearable ECG monitoring.

## 3. Biomimetics

Before systematically reviewing existing research on bionic electrodes, this framework is based on four fundamental compatibility issues that naturally exist between human skin and flexible ECG electrodes: mismatch in geometric morphology, mismatch in ionic transport pathways, mismatch in the physicochemical properties of materials, and mismatch in dynamic mechanical deformation. Four types of single-scale fundamental bionic designs have been derived to address each of these four individual issues. However, in actual dynamic monitoring, multiple mismatch issues often occur simultaneously; improvements in a single dimension are insufficient to meet the requirements for high-quality, long-term signal acquisition. Consequently, this paper further proposes the concept of ‘functional biomimicry’, which integrates multi-scale design. This has led to the formation of a classification framework comprising five dimensions—structure, interface, materials, mechanics and function—ranging from the surface to the interior, and from microscopic morphology to system integration.

### 3.1. Structural Biomimetics

During the actual wearing and application of flexible electrodes, problems such as slipping and falling off often occur, which seriously affect the stability and accuracy of potential data acquisition. At the same time, to achieve long-term and high-precision bioelectrical signal monitoring, the electrode needs to meet the requirements of long-term wearing. Therefore, its breathability and comfort have become key indicators that must be focused on in the design process. As an effective optimization strategy, structural biomimetics can significantly improve the comprehensive performance of electrodes by imitating and modifying the special structures of organisms in nature, providing a feasible path for solving the above problems [35]. Specifically, the application of structural biomimetics in electrode design is mainly reflected in the following aspects: simulating the micro-nano rough structure of human skin to form a closer fit between the electrode and the skin, increasing the actual contact area, which not only effectively enhances the anti-slip ability of the electrode but also improves the comfort during wearing; drawing on the adhesion mechanism of biological structures, such as octopus suckers and gecko setae, using van der Waals forces and capillary intermolecular forces at the micro-nanoscale to achieve physical adhesion between the electrode and the skin without chemical adhesives, avoiding skin irritation caused by chemical substances; and imitating the porous structure of sponges to construct a three-dimensional interconnected porous network, which, while ensuring the flexibility and conductivity of the electrode, forms effective air circulation channels and greatly improves the breathability of the electrode.

#### 3.1.1. Anti-Slip

Although ultra-thin flexible electrodes can achieve close contact with the dynamically curved surface of the human body relying on their low bending stiffness [36], air gaps are likely to form at the skin–electrode interface. In addition, electrodes with a microscale thickness, due to the mismatch in mechanical properties with the skin interface, generate motion artifacts under shear force or bending deformation, which seriously interfere with signal acquisition. The dual-functional design concept enhances interface compliance through the water-assisted capillary driving effect by endowing the electrode with hydrophilic–hydrophobic amphiphilicity, realizing gap-free close fitting. The reasonable selection of hydrophilic and hydrophobic materials and the layout of the amphiphilic structure further improve the electrode fitting degree, structural stability, and waterproof performance, providing a core solution for solving the problem of efficient fitting between the electrode and the skin.

**Figure 3:**
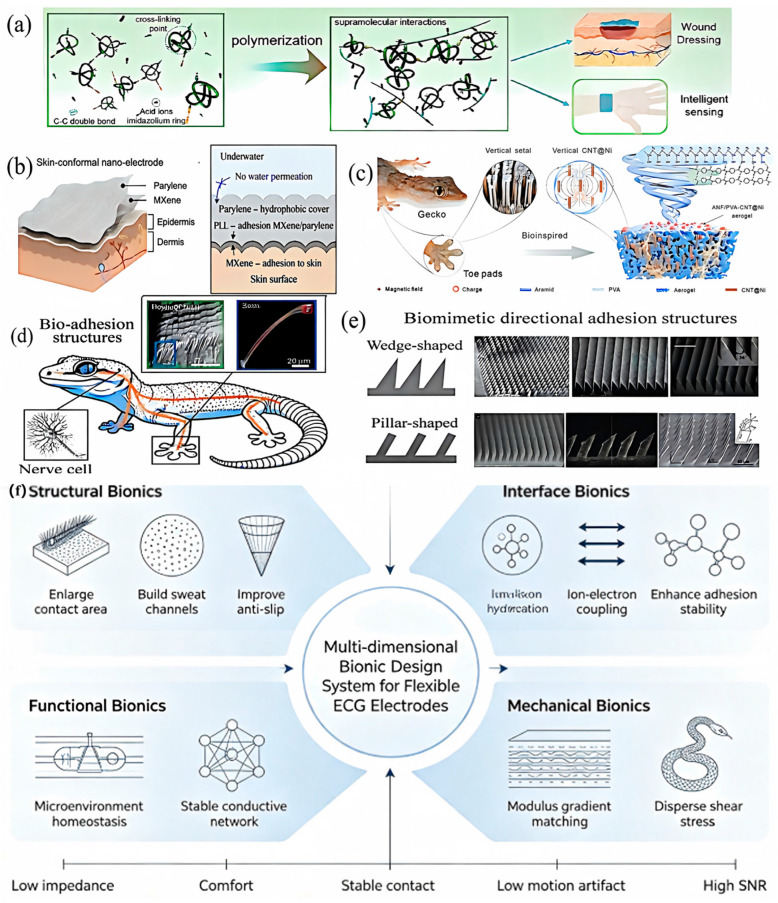
(**a**) Synthetic mechanism of CysgeAL [2]. Copyright 2024 ACS Appl Mater Interfaces. (**b**) A schematic diagram of skin-adhered nanoelectrodes, and a schematic diagram of waterproof skin-adhered nanoelectrodes based on amphiphilicity [24]. Copyright 2025 OPEN ACCESS ACS. (**c**) Schematic diagram of the vertical bristle structure of gecko toes and the corresponding microstructure [37]. Copyright 2025 Funk. (**d**) Microstructure and sensing of actual gecko toes [38]. Copyright 2024 OPEN ACCESS Sensors and Actuators A: Physical. (**e**) Common biomimetic structures of directional dry adhesives: wedge shape [37]. Copyright 2024 OPEN ACCESS Sensors and Actuators A: Physical. (**f**) Schematic diagram of bionic electrodes.

In terms of material selection, hydrophilic materials (such as Maxine) can further improve the fitting degree between the electrode and the skin through strong hydrophilic and polar interactions with the skin surface (Figure 3a), while hydrophobic materials can provide good structural integrity and chemical stability for the photosensitive film of the electrode, effectively resisting the damage of the external environment to the performance of the photosensitive film [2]. Through the amphiphilic structure design with the hydrophilic surface facing the skin and the hydrophobic surface facing outward, it can effectively block the direct penetration of water into the skin–electrode interface, endow the electrode with excellent waterproof performance, and ensure its stable operation in a humid environment (Figure 3b). Benefiting from its extremely low bending stiffness, the ultra-thin flexible electrode can form close contact with the dynamically changing curved surface of the human body, thereby firmly adhering to the skin, laying a solid foundation for the stable and efficient acquisition of bioelectrical signals [24].

To further optimize the skin adaptability of the electrode, researchers have innovatively proposed a dual-functional design idea that enables the electrode to have both hydrophilicity and hydrophobicity. This design utilizes the water-assisted capillary driving effect to promote the electrode to smoothly fit the human skin surface during the transfer process, significantly enhancing the high compliance of the skin–electrode interface, and ultimately achieving the ideal application effect of no air gaps, close physical contact, and excellent skin adhesion [24].

The micro- and nanoscale array morphology of the vertical bristles on a gecko’s feet, together with their shovel-like terminal topology, serves as the prototypical biomimetic structure for dry anti-slip electrodes. By utilizing the cumulative van der Waals adhesion generated through the superposition of high-density microunits, this approach provides a structural design concept for adhesive-free skin-adhering electrodes (Figure 3c). Inspired by this vertical fiber topology, the researchers fabricated a vertical aerogel based on aramid nanofibers, in which the magnetic carbon nanotube clusters (Cantini) maintain a vertical alignment under the combined action of magnetic torque and magnetic attraction; the ordered vertical microstructure significantly increases the number of effective contact sites with the skin. Furthermore, following optimization of the charge distribution, the amplitude of the device’s sensing signal increased by 64%. High-temperature cross-linking curing enables this vertical porous topology to be maintained stably; the structure does not collapse under a large strain of 80%, and its compressive strength reaches 680 kPa. When this vertical aerogel is fabricated into a flexible sensing electrode, the signal recognition accuracy reaches 98.2%, demonstrating the beneficial effect of the gecko-inspired hair structure on the electrode’s anti-slip properties and signal acquisition performance [37].

Relevant studies have confirmed that relying solely on van der Waals forces, a weak interaction, the synergistic force generated by the specific density of gecko setae, the area of toe pads, and the unique shovel-like structure at the end of each seta can form an adhesive force sufficient to support its own weight at the animal scale [38]. Furthermore, from a structural and geometric analysis, the gecko’s setae exhibit a gradually varying cross-sectional morphology from base to tip, which is adapted to the multiscale rough texture of the skin, thereby maximizing the microcontact area (Figure 3d). Further validation via finite element analysis demonstrated that this gradient microstructure disperses local stresses at the contact interface, preventing fracture failure during repeated attachment and detachment cycles (see Section 3.4 for the specific mechanism of action), thereby ensuring the adhesion stability of the electrode during long-term wear [39,40] (Figure 3e).

#### 3.1.2. Breathability

**Figure 4:**
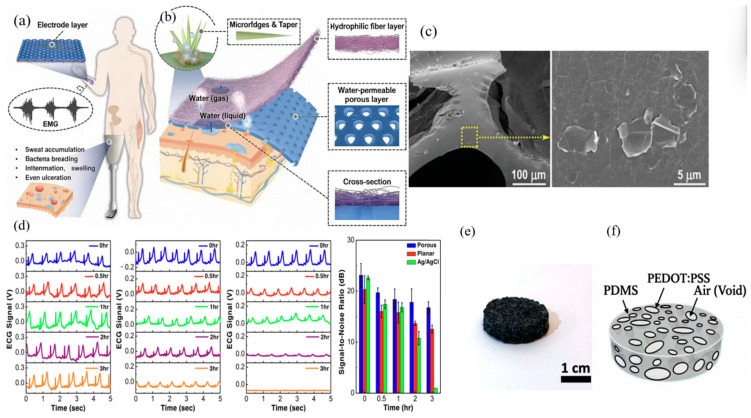
(**a**) Biomimetic epidermal electronics model [25]. Copyright 2022 OPEN ACCESS ACS. (**b**) Cactus cluster spines and trichome model [25]. Copyright 2022 OPEN ACCESS ACS. (**c**) Biomimetic sponge microstructure [26]. Copyright 2025 OPEN ACCESS Horas. (**d**) ECG signal measurement using porous PEDOT:PSS/PDMS electrodes; Planar PEDOT:PSS electrodes; Commercial Ag/AgCl electrodes; SNR comparison among the three different electrodes [26]. Copyright 2022 OPEN ACCESS ACS. (**e**) Photograph of the sponge electrode [26]. Copyright 2022 OPEN ACCESS ACS. (**f**) Schematic diagram of the sponge electrode structure [26]. Copyright 2022 OPEN ACCESS ACS.

The fog collection and directional transport mechanism formed by the conical spines and hair cluster structures of cactus stems provides an innovative biomimetic idea for improving the breathability of flexible electrodes (Figure 4a). Inspired by this, researchers have developed a biomimetic, flexible epidermal electronic device with both high gas permeability and unidirectional water transport capability [41], which is composed of three layers: a water-permeable porous layer (WPL), a hydrophilic fiber layer (HFL), and an electrode layer (ECC) [42].

This device utilizes a cactus-inspired gradient conical pore design to form a through-channel for water and vapor transport, delivering excellent sweat drainage and gas exchange capabilities. Experimental results indicate that this asymmetric conical pore array enables rapid unidirectional fluid transport in 1.09 s and anti-gravity water transport in 2.50 s, with the overall gas permeability of the electrode reaching 3551.63 g·m^−2^·d^−1^ and that of the porous fiber substrate increasing to 3795.38 g·m^−2^·d^−1^ (Figure 4b). Under rigorous ECG monitoring conditions—involving profuse sweating for 5 h and prolonged wear for 7 days—the conical through-hole design continuously drains surface moisture and prevents stratum corneum edema, whilst maintaining a stable ECG SNR of over 56 dB; its performance is far superior to that of sealed flat-surface structures and commercially available Ag/AgCl gel electrodes [25]. However, during prolonged wear, oils from the skin can easily become trapped in the narrow conical pores (with a skin-side pore diameter of just 30 μm), and the accumulation of fine impurities gradually blocks the water-delivery channels. As the duration of wear increases, the device’s breathability and water-conducting capabilities continue to decline, with a marked drop in performance observed towards the end of the 7-day continuous monitoring period.

The cactus (Opuntia microdasys microdosa) has evolved an efficient fog collection system, and the realization of this unique function benefits from the uniformly distributed conical spines and hair cluster structures on its stems. Many previous related studies have fully confirmed that cactus spines can efficiently collect tiny droplets in the air, and drive the directional transport of droplets with the help of the Laplace pressure gradient and the difference in surface free energy formed by the microrib structure on the surface of the conical spines; the collected droplets are immediately absorbed by the hairs on the stem surface and complete the circular transport process in the cactus body. The application of this unique structure of cactus in flexible electrode design shows significant advantages in improving electrode breathability and comprehensive performance, providing a new idea for solving the breathability problem of flexible electrodes during long-term wearing. The three-dimensional (3D) interconnected porous structure of sponges endows them with excellent mechanical deformation capacity and specific surface area advantages, providing core support for the design of breathability and mechanical durability of flexible electrodes (Figure 4c). Electrodes prepared with sponges as skeletons belong to a 3D conductive network structure, which not only provide efficient channels for electron and ion transport and reduce the overall resistance but also achieve high ion adsorption capacity and high active material loading through the high specific surface area, promote charge transfer at the electrode–electrolyte interface, and shorten the ion diffusion path, thus having broad application prospects in electrochemical energy storage and conversion systems (Figure 4d). In the field of wearable electrodes, the porous structure of sponges can realize efficient air circulation and rapid evaporation of moisture on the skin surface, effectively reducing the accumulation of heat and sweat during wearing, and meeting the wearing needs of special scenarios such as long-term physical activity. The 3D sponge electrode achieves the integration of multiple characteristics, including low resistance, excellent mechanical flexibility, outstanding electrochemical performance, and good gas permeability, providing strong technical support for the research and development of soft biosensors and flexible energy devices [26] (Figure 4e). The sponge structure has interconnected porous characteristics and good flexible deformation ability (Figure 4e). This unique structure enables it to effectively absorb and redistribute the stress applied to it when subjected to mechanical loads. Therefore, flexible electrodes prepared with sponges as skeletons can significantly reduce the risk of structural damage or performance failure of conductive materials when subjected to complex mechanical deformations such as bending, twisting, and stretching [43], thereby endowing the electrodes with good mechanical durability and extending their service life. At the same time, the porous structure makes the specific surface area of sponge electrodes much higher than that of corresponding bulk electrodes, a characteristic that is conducive to improving the electrical conductivity and sensing sensitivity of electrodes [44]. In addition, flexible electrodes based on sponge materials also show unique advantages in the wearable field: their porous structure is conducive to air circulation and evaporation of moisture on the skin surface, which can effectively reduce the accumulation of heat and sweat during wearing. This is crucial for meeting the wearing needs in special scenarios such as long-term physical activity and significantly improves the user experience of wearable devices. However, the three-dimensional porous structure of sponge materials has inherent structural defects; the disordered, interlaced arrangement of its micropores lacks uniformity, and the pore sizes are highly random. This tends to result in an uneven distribution of contact stress at the electrode interface, and following prolonged and repeated deformation, localized pore collapse and structural loosening are likely to occur. At the same time, the porous, open-work structure reduces the overall structural density of the electrode, making it highly prone to slight warping at the edges and a decline in interface adhesion consistency, which in turn affects the stability and repeatability of ECG signal acquisition.

In terms of anti-slip performance, structural biomimetics have achieved stable fitting between electrodes and the skin. The amphiphilic structure design eliminates air gaps at the interface, improving the fitting degree and waterproof performance; the gecko setae biomimetic structure enhances anti-slip ability and sensing performance by virtue of van der Waals forces; and the octopus sucker biomimetic design endows electrodes with high compliance, and its sensor array can realize programmable control of sensitivity, featuring excellent stability and anti-crosstalk capability, which is suitable for multi-scenario applications. In terms of breathability and mechanical durability, the cactus biomimetic structure enables electrodes to have both high breathability and rapid water transport capability, ensuring signal stability during long-term wearing; the porous sponge structure constructs an efficient conductive network, improving sensing performance, while promoting air circulation and sweat evaporation, enhancing the mechanical durability of electrodes and providing support for the research and development of related devices. In summary, structural biomimetics has realized the synergistic improvement of multiple performances of flexible electrodes, addressed the intrinsic drawbacks of conventional electrodes, and provided important support for the research and development and industrialization of high-performance flexible electrodes.

### 3.2. Interface Biomimetics

During the fitting process of flexible electrodes with the skin, problems such as excessively high interfacial impedance and unstable interface integration seriously affect the transmission efficiency and stability of bioelectrical signals, restricting the monitoring accuracy and application effect of the electrodes. Interface biomimetic technology, by simulating the ion transport characteristics and biomolecular adhesion mechanisms of biological systems, constructs an optimized electrode–skin interface, solving the above key problems from three dimensions: reducing interfacial impedance, stabilizing interface combination, and improving long-term stability, thus laying a foundation for the high-performance application of flexible electrodes.

#### 3.2.1. Ionic–Biomimetic Interface

In the field of bioelectronics, the mismatch of charge carriers at the biointerface is a key bottleneck restricting device performance, and the development of ion electronics has provided an effective solution to this problem. Taking ions as charge carriers, the ionic–biomimetic interface achieves intrinsic compatibility with biological systems after integrating electronic functional components, becoming a versatile platform with multiple functions [45]. This interface can realize direct coupling with ion flows in the organism, construct transduction pathways similar to the native ionic signal conduction mechanism, and significantly improve the sensitivity and accuracy of electrophysiological sensing by reducing interfacial impedance and improving signal transmission quality, which is widely applicable to fields such as ECG, EMG, electrooculography (EOG), and brain wave monitoring.

In addition, the dynamic cross-linking effect between ions can real-time regulate the polymer network, endowing the material with characteristics such as self-healing, adjustable stiffness, and mechanical adaptability, enabling it to maintain close and stable long-term contact with soft biological tissues. In advanced systems, reversible ionic coordination or cross-linking can realize the physiological/environmental signal-responsive regulation of adhesion, endowing the electrode with programmable adaptive functions [46], which further expands the application scenarios of flexible electrodes [47]. In the field of bioelectronic applications, ion electronics achieves intrinsic compatibility with biological systems by using ions as charge carriers and integrating electronic functional components, thereby positioning the ionic–biomimetic interface as a versatile platform with multiple functions [45]. Functional systems containing ionic materials help realize direct coupling with ion flows in the organism, support ionic signal transduction pathways very similar to the native ionic signal conduction mechanism, and provide a guarantee for the accurate acquisition of bioelectrical signals. By reducing interfacial impedance and improving signal transmission quality, such ionic–biomimetic interfaces exhibit significantly enhanced performance in electrophysiological sensing applications, which can effectively improve the sensitivity and accuracy of signal detection. In addition to excellent sensing functions, the interaction between ions can also dynamically regulate the polymer network in real time through dynamic cross-linking, endowing the material with key properties such as self-healing ability, adjustable stiffness, and good mechanical adaptability, enabling it to maintain close and stable long-term contact with soft biological tissues and meet the long-term use needs of bioelectronic devices [27].

#### 3.2.2. Skin–Electrode Ionic–Electronic Interface

**Figure 5:**
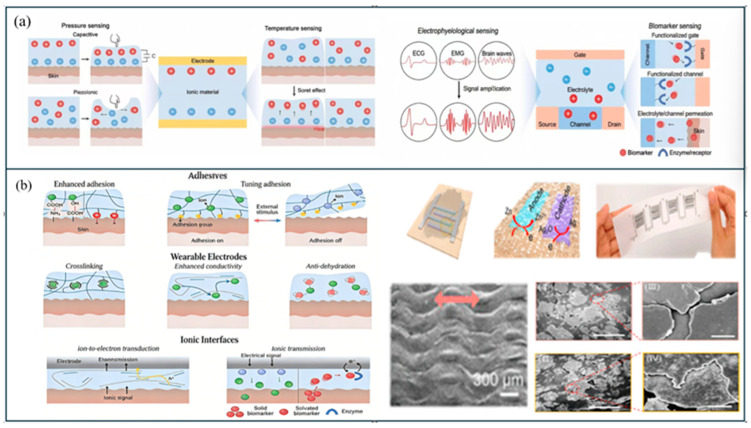
(**a**) Ionic–electronic sensors detect pressure through capacitance (EDL modulation) or piezo-ionic (ionic polarization) mechanisms, and detect temperature through ion thermodiffusion driven by the Sorbet effect. Sensors based on organic electrolyte transistors (OECTs) amplify low-amplitude electrophysiological signals (ECG, electromyography, brain waves) and detect biomarkers through ion penetration or specific molecular interactions of the gate or channel, providing multimodal biosensing capabilities [27]. Copyright 2025 OPEN ACCESS Sci. (**b**) Structural design and micromorphology of flexible electrodes [27]. Copyright 2025 OPEN ACCESS Sci.

The ionic–electronic interface formed between the skin and the electrode is the core carrier for bioelectrical signal acquisition. Its capacitance is positively correlated with the contact area of the electrode–electrolyte interface, and there is no obvious electrochemical reaction during the electron-ion separation process under low voltage (Figure 5a). The natural internal ion exchange capacity and waterproof protection characteristics of human skin provide an ideal design idea for overcoming the stability and compatibility defects of synthetic conductive gel sensors. The skin–electrode mechanical sensor (SEMS) developed based on this uses the skin as a natural ionic material, realizing the synchronous measurement of physiological signals and external mechanical stimuli. The sensor is composed of the skin, a sensing electrode (SE) with a microstructure surface, and a highly conformable counter electrode (CE). It is fixed by applying a basic pressure of about 5 kPa through a transparent and breathable health-care film, without the need for a synthetic ionic gel/hydrogel, and its structure is far superior to that of traditional layered complex electronic skin (Figure 5b). Researchers also explored the key geometric features for improving pressure sensitivity by utilizing structural instability through mechanical analysis and simulation, which not only accurately predicted the electrode deformation response but also provided important theoretical guidance for the optimal design of the electrode surface microstructure.

#### 3.2.3. Stable Interface: Mussel Byssus–Dopamine Biomimetic Interface

Appropriate adhesion is the key for skin-like electrodes to achieve high-precision signal monitoring. The strong interaction between dopamine units of mussel byssus and solid surfaces serves as the core biomimetic basis for the design of artificial adhesive materials [48], while its long-overlooked hierarchical porous structure provides a new idea for improving adhesion stability and electrode damage resistance. The micro/nanoscale pores of mussel byssus can expel moisture on humid surfaces, avoiding the interference of the hydration layer on the adhesion of dopamine units [49]; combining the ultra-fast water/ion transport characteristics of carbon nanotube nanochannels [50] and the quantum-confined superfluid theory [51], researchers proposed the idea of constructing a similar pore structure for flexible skin-like ionic electrodes, maintaining conductivity through ultra-fast ion transport before electrode damage healing. Based on this idea, researchers utilized the solid-phase molecular self-assembly (SPMSA) strategy [47] to construct a damage-resistant, adhesive, flexible skin-like ionic electrode with a hierarchical pore structure mimicking mussel byssus through the electrostatic interactions and hydrogen bonding between three polyelectrolytes, introducing conductive ions and three-dimensional nanoscale channels into the film to balance the elasticity and viscosity of the film. Even if the electrode has cuts and fracture damage, as long as there are still small connected parts in the damaged area, ion conduction can remain constant, realizing stable real-time signal measurement. This has for the first time confirmed the possibility of manufacturing damage-resistant, high-stability flexible skin-like electrodes for human–computer interaction, providing a new technical path for the construction of stable electrode interfaces [52]. For the skin–electrode ionic–electronic interface, when a small voltage is applied, electrons and ions at the interface will separate without obvious electrochemical reactions, and its capacitance is positively correlated with the interface contact area between the electrode and the electrolyte. As a natural biological tissue, human skin not only has internal ion exchange capacity, which can form good interface coupling with ionic electrodes, but also has a waterproof protective outer layer, which can effectively block the interference of the external environment on the interface. This unique physiological characteristic makes it an ideal choice to overcome the great challenges such as poor stability and insufficient compatibility faced by sensors based on synthetic conductive gels in practical applications, providing natural design inspiration for the development of high-performance skin–electrode interfaces.

#### 3.2.4. Long-Term Operational Stability

Reducing the thickness of electrode materials to lower the Young’s modulus is an effective method to improve the flexibility of dry electrodes and the quality of signal acquisition [53], but the relative movement at the skin–electrode interface still generates interference signals during long-term dynamic monitoring. Therefore, constructing a stable interfacial bonding for high-fidelity signals is the core to achieving long-term stable monitoring [28]. At present, the main methods to enhance interfacial bonding force include chemical modification to form intermolecular attractive forces [54] and biomimetic microstructures to improve physical adhesion [54,55]. However, these methods are prone to causing skin irritation, and high-adhesion electrodes are prone to irreversible deformation and damage when detached, making it difficult to achieve repeated use. Currently, few reports on bioelectrodes simultaneously possess long-term (>3 days) stability, dynamic environmental stability, reliable reusability, and low skin irritation. In response to this situation, researchers have developed dry electrodes based on biostable adhesive polymers (BAP) [56] and highly conductive silver nanowire (Agnes) networks, realizing on-demand switching of the interfacial adhesion state: when triggered by skin temperature, the electrode exhibits high flexibility and strong adhesion, which can closely fit the skin under humid conditions; after treatment with cold water/ice packs, the modulus increases significantly and the adhesion decreases, allowing easy detachment without skin irritation. Compared with triggering methods such as pH, humidity [38], and magnetism, the temperature control method is more practical. Meanwhile, the wrinkled microstructures on the electrode surface effectively extend the stretchability of the Agnes layer, resisting irreversible damage to the conductive network caused by epidermal deformation and electrode detachment. Experimental results show that compared with commercial Ag/AgCl gel electrodes, BAP electrodes can collect higher-quality bioelectrical signals under strong interference conditions, such as body movement and sweat, and can still maintain stable signal acquisition performance after 7 consecutive days of ECG monitoring or 10 repeated uses. Reusable surface ECG electrodes can adopt the temperature-sensitive biomimetic adhesion system. Biostable polymers matched with silver nanoconductive networks provide sufficient viscosity at body temperature and can be peeled off without irritation under low temperature. Wrinkled microstructures on the surface protect conductive layers, and stable ECG signals can be collected after 7 consecutive days or 10 repeated uses, completely overcoming the allergy and single-use defects of traditional gel electrodes.

#### 3.2.5. Summary

In terms of reducing interfacial impedance, the ionic–biomimetic interface uses ions as charge carriers to achieve compatibility with biological systems. It can directly couple ion flows in the organism, reduce impedance, improve the accuracy of electrophysiological sensing, and is suitable for various monitoring scenarios; dynamic ionic cross-linking endows the material with characteristics such as self-healing, ensuring close contact with biological tissues. The skin–electrode ionic–electronic interface leverages the natural characteristics of the skin to avoid the defects of synthetic gels, featuring a simple sensor structure, and its sensitivity can be improved through microstructure optimization. In terms of stable interface construction, the mussel byssus–dopamine biomimetic interface can improve the stability and damage resistance of electrodes. Its dopamine units form a strong adhesion effect, and the hierarchical porous structure avoids the interference of the hydration layer; the biomimetic electrode based on this structure can maintain ion conduction when damaged, providing a new path for the preparation of high-stability electrodes. In terms of improving long-term stability, the biomimetic dry electrode can switch the adhesion state through skin temperature, which has the advantages of flexibility, strong adhesion, low irritation, and high practicality; the wrinkled microstructures protect the conductive network. Compared with commercial electrodes, it has higher signal quality under strong interference and can perform continuous monitoring for 7 days or be reused 10 times. In summary, interface biomimetic technology has realized the synergistic optimization of the interface performance of flexible electrodes, solved the core pain points of traditional electrodes, and provided important support for the research, development, and application of high-performance flexible electrodes.

### 3.3. Biomimetic Materials

As the theoretical foundation and core approach for the design of bionic electrodes, material bionics is fundamentally based on the systematic simulation of the basic components, microstructures, and key physicochemical properties of human biological tissues. It aims to address a series of critical bottlenecks associated with traditional electrode materials, such as poor interfacial compatibility with human skin or myocardial tissue, unstable bioelectrical signal acquisition, and insufficient long-term wearing comfort. Through this approach, high-level matching and dynamic adaptation between electrode materials and biological tissues in terms of physical properties, chemical characteristics, and functional mechanisms are ultimately achieved [57]. The mainstream material systems currently used for bionic electrodes include elastic conductive polymers, nanocomposite conductive materials, and conductive hydrogels.

#### 3.3.1. Flexible Conductive Polymers

This bionic prototype, featuring an elastic conductive polymer core, draws inspiration from human skin, fishing nets, and honeycomb structures. By replicating the functional characteristics of these natural structures, the material’s performance is optimized: mimicking human skin, it utilizes its flexibility, stretchability, and sensory properties, whilst replicating micro- and nanoscale wrinkled structures to enhance the material’s adaptability to deformation and its sensing capabilities. It adheres to the skin to collect stable ECG signals. Based on an interwoven porous configuration, an interpenetrating network of elastic matrix and conductive fillers is constructed to enhance electrical conductivity stability under electrode deformation. Relying on a multi-level ordered porous structure, external forces are dispersed, and fracture energy is dissipated; combined with a conductive skeleton, this enhances elastic recovery and electrical conductivity durability, meeting the requirements for long-term ECG monitoring. Common elastic polymers include traditional elastomers, hydrogels, and ionic gels.

**Figure 6:**
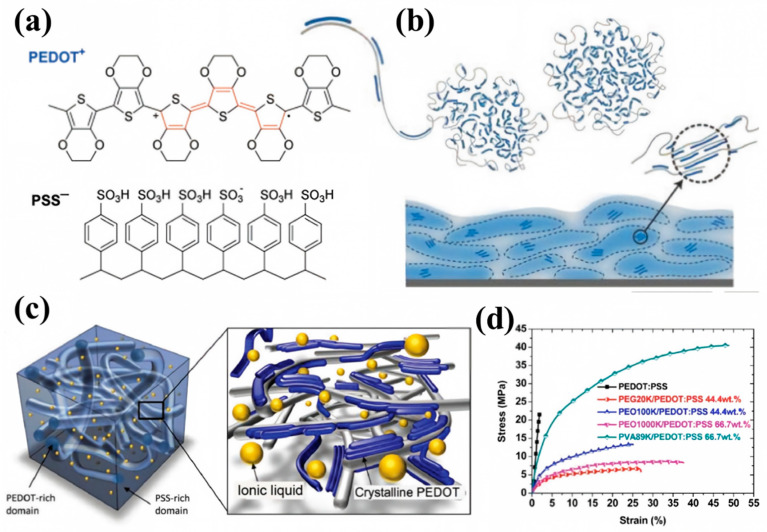
(**a**) Chemical structure of PEDOT:PSS [58]. (**b**) PEDOT:PSS formation of colloidal gel particles when dispersed in water and microstructure of the resulting films with PSS-rich domains (gray) and PEDOT:PSS-rich domains (blue). (**c**) Enhancing the stretchability of PEDOT:PSS by blending with ionic liquid plasticizers [58]. (**d**) Adapted with permission from CC BY-NC 4.0 license [58]. Copyright 2025 Adv. Mater.

Traditional elastomers are inherently insulating and require the addition of conductive fillers to impart electrical conductivity. Examples include metallic nanoparticles (such as silver nanowires/nanoparticles, copper nanowires and gold nanosheets), carbon-based fillers such as carbon nanotubes (CNT), carbon black [58], graphene (GR), reduced graphene oxide [17], Menes, and intrinsically conductive polymers such as poly(3,4-ethylenedioxythiophene):poly(styrene sulphate (PEDOT:PSS), polypyrrole (PPy), and polyaniline (PANI). The conventional elastomer PDMS is a silicone elastomer characterized by good elasticity, high transparency, chemical and thermal stability, simple processing steps (Figure 6a), and biocompatibility with human tissues and cells. Conductive materials are typically dissolved in organic solvents to form a homogeneous solution, which is then uniformly deposited onto the PDMS surface, followed by solvent evaporation to obtain a flexible conductor. There are various solution-based deposition methods, including spraying [59], drop casting [60], spin coating, and dip coating [61]. The spin-coating method is widely used because it produces uniform coatings, and the coating thickness can be controlled by the rotation speed. A photoreactive conductive ink containing PEDOT:PSS within a carrier protein matrix is spin-coated onto methacrylate-functionalised PDMS. The ink is then exposed to ultraviolet (UV) light through a photomask, causing cross-linking in the exposed areas to yield the corresponding elastic conductive polymer. PEDOT:PSS is a conductive polyelectrolyte composite obtained by first synthesizing PSS, followed by the aqueous oxidative polymerization of PEDOT using a strong oxidizing agent (sodium or ammonium persulphate) in the presence of the PSS matrix. It exhibits high electrical conductivity and high light transmittance, and can be processed in water; however, the elongation at break of PEDOT:PSS is limited to approximately 10%, meaning that when used in flexible electrodes for motion monitoring, the electrodes are prone to deformation (Figure 6b). Inspired by biological skin, researchers mixed PEDOT:PSS with low-molecular-weight plasticisers, effectively reducing the interactions between polymer chains [62]. This allows PEDOT:PSS to be stretched by approximately 50%, increasing its free volume and thereby lowering its elastic modulus to a value similar to that of human tissue, thus enhancing the material’s tensile properties. Common plasticisers for PEDOT:PSS include xylitol, glycerol, Triton X-100 (a non-ionic surfactant derived from ethylene glycol) and ionic liquids (salts with a melting point below 100 °C). Another method for improving the stretchability of PEDOT:PSS is to blend it with polymers; for example, the addition of the amphiphilic block copolymer PDMS-b-PEO enhances the miscibility of water-based PEDOT:PSS in the PDMS precursor [63] (Figure 6d). Experiments revealed that the addition of 30 wt.% PDMS-b-PEO yielded the optimal balance between electrical conductivity and tensile strength (Figure 6c). Surprisingly, its electrical conductivity was identical to that of undoped PEDOT:PSS (≈0.3 S/cm), and it could be stretched by up to 75% [64]. PEDOT:PSS can also be blended with the hydrophobic rubber-like poly (butyl acrylate-styrene) (P(BA-r-St)) latex elastomer to achieve an elongation of 97% and a conductivity of 63 S/cm. Studies have shown that small-molecule plasticizers insert themselves into the interior of PEDOT:PSS composites via hydrogen bonding and electrostatic interactions, thereby weakening the strong electrostatic bonds between PEDOT^+^ and PSS^−^. This induces microscopic phase separation within the system, promoting the aggregation and crystallization of PEDOT conductive chains to form a continuous conductive network [65]. Concurrently, the plasticizer increases the free volume of the PSS segments, enhancing the film’s flexibility and film-forming properties. Upon mixing with the polymer, PEDOT:PSS induces nanophase separation through interfacial compatibility, resulting in a composite structure comprising an elastic matrix and a conductive network. The synergistic interaction between the molecular plasticizing effect and multiple energy dissipation mechanisms improves the film’s ductility and crack resistance.

Consequently, when PEDOT:PSS is blended with PDMS-b-PEO, the hydrophilic PEO segments in PDMS-b-PEO interact with PEDOT:PSS via hydrogen bonding and electrostatic interactions, thereby reducing the strong interactions between PEDOT and PSS and alleviating the rigidity and brittleness caused by excessive cross-linking in pure PEDOT:PSS films [66]. Hydrophobic, flexible PDMS segments form a continuous elastic matrix, providing the composite system with high ductility. During tensile loading, the PEDOT:PSS conductive domains within the soft matrix undergo slippage, orientation, and reversible reorganization, thereby preventing stress concentration and rapid crack propagation. This significantly enhances the film’s tensile strength and toughness whilst maintaining the conductive pathways. Both approaches achieve optimization of the mechanical tensile properties and electrical performance of PEDOT:PSS.

#### 3.3.2. Nanocomposite Conductive Materials

Nanocomposite conductive materials are functional materials with excellent electrical conductivity, produced by incorporating nanoscale conductive fillers into a matrix of polymers, inorganic materials, or similar substances, and then combining them through specific preparation processes. Depending on the type of conductive filler used, they can be classified into four categories: carbon-based nanocomposite conductive materials, metal-based nanocomposite conductive materials, polymer-based nanocomposite conductive materials, and composite filler-type nanoconductive materials [67]. Carbon-based conductive nanocomposite materials utilize carbon nanotubes (CNTs), reduced graphene oxide (RGO), nanographite, and carbon black as conductive fillers. They offer excellent conductivity, good biocompatibility, light weight and high flexibility, and are currently among the most widely used nanomaterials in the field of ECG electrodes. For example [30], graphene has a theoretical electrical conductivity of up to 10^6^ S/m and a specific surface area of up to 2630 m^2^/g, which effectively enhances the material’s electrical conductivity and contact area with the skin; carbon nanotubes possess a unique one-dimensional nanostructure that enables the formation of a continuous conductive network within the matrix, significantly reducing the material’s volume resistivity. Currently, researchers are using techniques such as redox methods and in situ polymerisation to composite graphene and carbon nanotubes with flexible polymers (such as polydimethylsiloxane and polyimide), thereby producing carbon-based nanocomposite conductive materials that exhibit excellent flexibility and good electrical conductivity [68]. Metal-based nanocomposite conductive materials utilize metal particles such as silver nanoparticles, gold nanoparticles and copper nanoparticles as conductive fillers. Whilst they offer excellent conductivity and rapid signal response, their long-term application is limited by issues such as poor biocompatibility, susceptibility to oxidation and insufficient flexibility. Researchers have improved the biocompatibility and oxidation resistance of metal nanoparticles through methods such as silane coupling agent modification and polymer coating. By combining these with flexible matrices, they have produced metal-based nanocomposite conductive materials that combine high conductivity with flexibility. Polymer-based nanocomposite conductive materials use conductive polymers as the matrix, modified by the incorporation of nanofillers, and offer advantages such as good flexibility, excellent biocompatibility, and ease of processing and forming. Conductive polymers possess inherent conductivity; by incorporating nanofillers to form a continuous conductive network, the material’s electrical conductivity and mechanical stability are further enhanced.

The biomimetic design of nanocomposite conductive materials is primarily reflected in two aspects: firstly, the selection of flexible matrix materials that mimic the suppleness and elasticity of human skin. Secondly, the water content of hydrogels is similar to that of human skin, giving them good softness and conformability, enabling them to form a ‘hydrated bond’ with the skin, thereby reducing interfacial friction and irritation [69]. Thirdly, by modifying the materials to enhance their biocompatibility and mimic the biological properties of human skin, bioactive substances such as collagen and chitosan are coated onto the material surface. This improves compatibility between the material and skin cells, reduces inflammatory responses, and ensures comfort and safety during long-term wear [31].

#### 3.3.3. Conductive Hydrogels

**Figure 7:**
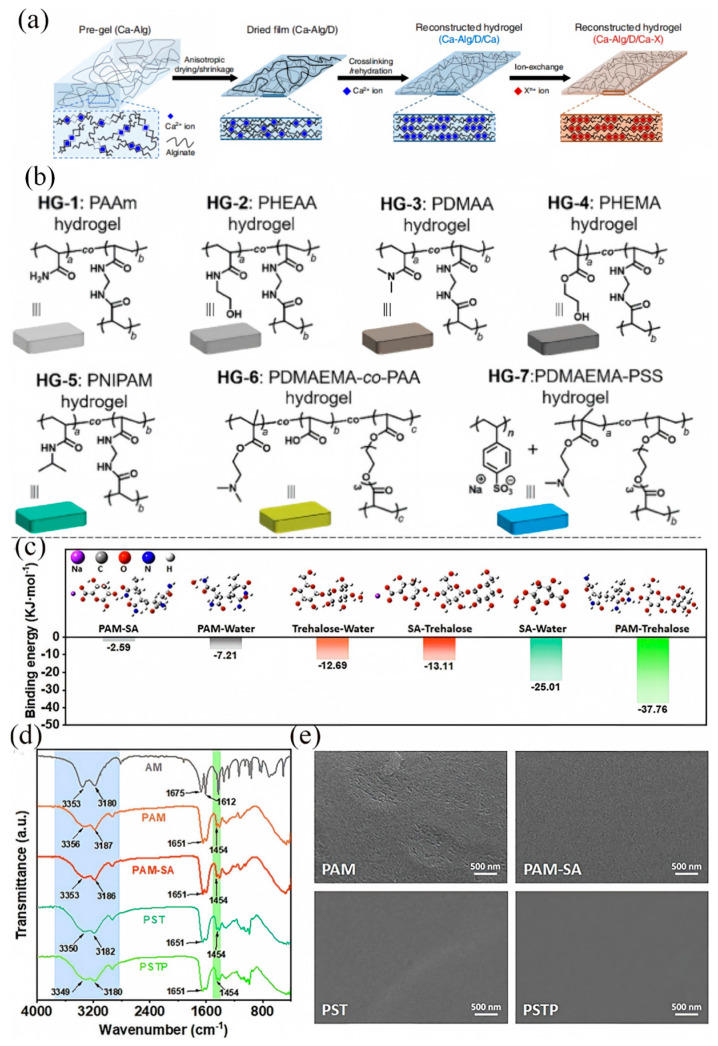
(**a**) Schematics of fabrication for reconstructed Alg hydrogel and mechanically robust aqueous supercapacitor [70]. (**b**) Construction of the hydrogel assembly process by using the PAA hydrogel as an adhesive layer in this work, as well as chemical structures of employed hydrogels (PAA hydrogel, HG-1, HG-2, HG-3, HG-4, HG-5, HG-6, HG-7) [71]. Copyright 2024 Small. (**c**) The interactions between SA, trehalose, water and PAM molecules [23]. Copyright 2025 OPEN ACCESS Chemical Engineering Journal. (**d**) FTIR spectra of AM, PAM, PAM-SA, PST and PSTP hydrogels [23]. Copyright 2025 OPEN ACCESS Journal of Materials Chemistry A. (**e**) SEM images of PAM, PAM-SA, PST, and PSTP hydrogels [77]. Copyright 2025 OPEN ACCESS Journal of Materials Chemistry A.

Conductive hydrogels consist of conductive materials and a cross-linked hydrogel polymer network; they are soft materials that combine a three-dimensional cross-linked network, high water content, and electrical conductivity, achieving conductivity through the conduction of ions, electrons, or a combination of both [70] (Figure 7a). Brown algae are an ancient group of marine plants that typically grow rootless on rocks, which has inspired scientists to investigate the adhesion mechanisms underlying their rootless growth. Inspired by the adhesion mechanism of brown algae [71], the researchers synthesized PSTP conductive hydrogels by dissolving acrylamide (AM) (Figure 7b), SA, and trehalas in a dispersed PEDOT:PSS solution using a one-pot thermal copolymerization method. Furthermore, repeated experiments have shown that PSTP hydrogels composed of 2% SA, 30% trehalose, and 1% PEDOT:PSS exhibit optimal adhesion [23] (Figure 7c,d). Multiple adhesion tests were conducted on this PSTP conductive hydrogel. After 60 repeated adhesion cycles, it still demonstrated satisfactory adhesive strength, and its ionic conductivity remained at 84% of its initial value [72]. Furthermore, the PSTP conductive hydrogel can withstand significant deformation; it does not suffer significant damage even under uniaxial tensile or torsional stress, demonstrating its excellent flexibility. This endows flexible wearable electrodes with superior adhesion and tensile properties [73].

Research indicates that the rootless growth of brown algae is attributed to their excreted adhesive compounds, which include calcium alginate gel, phycellin, and sulphated fucoidan. The hydroxyl groups abundant in the chlorotannins of brown algae facilitate their adhesion to substrates [74]. Based on this phenomenon, during the preparation of PSTP conductive hydrogels, the polyhydroxyl groups of SA exhibit a strong affinity for the nucleophilic groups on the PAM chains, establishing an effective network of intermolecular hydrogen bonds between SA and the PAM chains. The presence of these abundant hydroxyl and amide groups provides a large number of active sites, enabling the formation of reversible non-covalent interactions, such as hydrogen bonds and coordinate bonds, with various substrates [75]. These interactions are utilized as a marker for confirming the successful synthesis of PSTP hydrogels via Fourier-transform infrared (FTIR) spectroscopy.

When the PSTP hydrogel comes into contact with the skin, the numerous hydroxyl groups and amide bonds on the hydrogel surface form multiple hydrogen bonds and electrostatic interactions with the amide, amino, and hydroxyl groups on the skin surface [76]. The hydrogel can be removed from the skin surface provided that the applied external force exceeds the intermolecular forces between the hydrogel and the skin (approximately 3.75 N). When the hydrogel comes into contact with the skin again, hydrogen bonds and electrostatic interactions can form between the two, causing them to re-adhere. Consequently, a large number of amide, amino, and phenolic hydroxyl groups are retained within the hydrogel, enabling it to adhere to the skin for a prolonged period and repeatedly through hydrogen bonding and electrostatic interactions. The polymer chains between different substances within the hydrogel, due to electrostatic interactions and van der Waals forces between the different substances, have led to the successful construction of a three-dimensional PSTP hydrogel network [77]. The microstructures of the PAM, PAM-SA, PST, and PSTP hydrogels were observed using SEM (Figure 7e), which also revealed that the PSTP conductive hydrogel exhibits a dense three-dimensional structure. As a class of specialized functional materials tailored for biomedical applications, biomimetic electrode materials possess highly biomimetic properties—combining the soft texture of biological tissues, a high water content compatible with the body’s physiological environment, excellent biocompatibility with no significant cytotoxicity, and the core capability of stably conducting electrical signals [32]. Such biomimetic materials can establish highly efficient conductive pathways adapted to the internal biological environment. Not only do they enable the efficient transmission and precise regulation of electrical signals, but they also form a good fit with biological tissues, significantly reducing the body’s foreign body reaction and tissue damage. Suitable for a wide range of biomedical applications, they demonstrate unique advantages in fields such as wearable biosensors, cardiac pacemaker electrodes, neural regeneration scaffolds, and cellular electrophysiological regulation [78]. Currently, their application still faces challenges in balancing properties such as mechanical strength and adhesion; traditional fabrication methods struggle to fully exploit their biomimetic advantages. However, advanced manufacturing technologies such as 3D printing can precisely control their structure, fully unlocking the application potential of biomimetic materials and providing support for their clinical translation [44,79].

### 3.4. Biomimetic Mechanics

**Figure 8:**
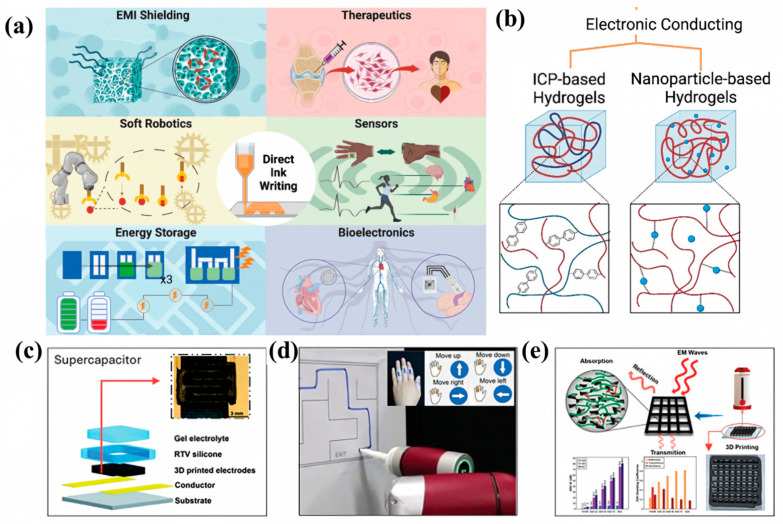
(**a**) Examples of different applications for DIW of conductive hydrogels include electromagnetic interference shielding, therapeutics, soft robotics, sensors, energy storage, and bioelectronics [80]. Copyright 2025 Advanced Functional Materials. (**b**) Conductive hydrogels can generally be categorized into electronically and ionically conductive types [80]. Copyright 2025 Advanced Functional Materials. (**c**) DIW printed five-layer micro supercapacitor using GO ink [80]. Copyright 2025 Advanced Functional Materials. (**d**) Wearable electronic skin made of PEDOT:PSS-PVA controlling an industrial robot [80]. Copyright 2025 Advanced Functional Materials. (**e**) EGO/TOCNF printed hydrogel was shown to be effective for EMI shielding [80]. Copyright 2025 Advanced Functional Materials.

Biological tissues possess unique mechanical properties; their elastic modulus, toughness, viscoelasticity, and stress–strain response have been optimized through long-term evolution to adapt to dynamic loads in physiological environments (such as limb movement, tissue peristalsis, and cardiac contractions), whilst maintaining mechanical compatibility with surrounding tissues to prevent tissue damage or electrode failure caused by mechanical mismatch [80]. The core objective of mechanical biomimicry is to design the structure, materials, and interface properties of electrodes by simulating the mechanical behavior of biological tissues, thereby enabling biomimetic electrodes to achieve mechanical compatibility with the human physiological environment (Figure 8a). This ensures structural stability during implantation or attachment whilst reducing mechanical stress between the electrode and the tissue, thereby enhancing the electrode’s long-term biocompatibility and operational reliability.

#### 3.4.1. Bionic Structures on Flexible Substrates

As the core component responsible for mechanical load-bearing, the structural design of the electrode substrate directly determines the overall mechanical properties of the electrode. Traditional commercial metal-based, thick-film rigid electrode substrates generally exceed 200 μm in thickness and have a modulus of elasticity ranging from several hundred kilopascals to megapascals, representing a mismatch of several orders of magnitude compared to the mechanical properties of human skin (1–100 kPa) and neural tissue (10–100 kPa). During deformation, rigid substrates are unable to expand and contract in synchronisation with the skin, resulting in enormous shear stresses at the interface. This makes the electrodes highly prone to curling and detachment, as well as fracture of the metal conductive layer. Prolonged wear also exerts continuous pressure on the skin, leading to irritation such as redness and indentations [81]. In response to these issues, this study proposes a ‘macroscopically flexible, microscopically biomimetic’ dual-scale design approach, resulting in the development of a biomimetic substrate that combines high flexibility with high ductility [82]. Ultra-thin flexible films with a thickness of 5–50 μm are used as the main substrate material, with materials such as PDMS, polyimide (PI), and hydrogels as the main substrate material. Their elastic modulus can be adjusted to 1–100 kPa, precisely matching the elastic modulus of human skin (1–100 kPa) and neural tissue (10–100 kPa), thereby preventing tissue compression injury caused by an excessively rigid substrate. At the microscale, by mimicking the interwoven structure of biological fibers, micro- and nanoscale grooves, grids, or honeycomb structures are created on the substrate surface, further enhancing the substrate’s flexibility and ductility [83]. By using photolithography to fabricate parallel grooved structures with a width of 10–50 μm and a depth of 5–20 μm on the surface of a PDMS substrate—mimicking the arrangement of collagen fibers in the dermis—the substrate’s elongation at break can be increased to 300–500% (Figure 8b), thereby meeting the demands of the skin’s maximum tensile deformation [84]. In contrast, the elongation at break of a smooth PDMS substrate of the same thickness, without microscopic grooves, is less than 100%, whilst that of traditional metal film substrates is even lower than 5%, making them highly prone to cracking and delamination under dynamic conditions. However, whilst ultrathin macroscopic substrates significantly enhance tensile strength, their reduced thickness also makes them highly susceptible to damage from external hard objects, thereby shortening the service life of the electrodes.

#### 3.4.2. Biomimetic Interface Mechanics

The mechanical properties of the interface between the electrode and biological tissue are key factors affecting the long-term stability of the electrode. Drawing on the adhesion mechanisms of biological tissue, the electrode surface is modified using biomimetic techniques to enhance the interfacial bonding strength between the electrode and the tissue, whilst simultaneously reducing the interfacial coefficient of friction and minimizing tissue damage [85]. For implantable electrodes, Gong et al. [86] demonstrated a one-step, catalyst-free chemical vapor deposition process capable of customizing Young’s modulus and geometry of carbon nanotube fibers, which facilitates the formation of a stable electrode–muscle interface. Tang et al. reported a multi-layered microfiber electrode with an adjustable elastic modulus, comprising a carbon nanotube core and a sodium alginate shell. Upon implantation, the hydrated sodium alginate softens the electrode, ensuring dynamic stability at the interface and reducing inflammatory responses, thereby enabling the monitoring of stable electrical signals from cortical neurons over a period of four weeks [87]. The surfaces of implantable electrodes are typically coated with biomimetic peptides (such as RGD peptides) or collagen, which mimic the composition and structure of the extracellular matrix. This promotes the adhesion and growth of tissue cells on the electrode surface, forming a stable interface bond, reducing interfacial stress and minimizing tissue fibrosis. Among these, implantable fiber electrodes have become a key area of research in biomedical engineering due to their capacity for continuous monitoring and their stability [88]. For skin-adhesive electrodes [89], a layer of biomimetic hydrogel is applied to the electrode surface to mimic the skin’s moisture-retaining properties, with the hydrogel’s water content maintained at 70–90%. Its adhesion strength reaches 1–5 N/cm^2^ (Figure 8c), enabling long-term adhesion of the electrode to the skin surface, whilst also offering good breathability and biocompatibility, thereby preventing skin allergies or inflammation (Figure 8d).

The adhesive strength of traditional electrode patches relies primarily on pressure-sensitive adhesive (PSA), a viscoelastic material that adheres firmly to a surface upon the application of light pressure. When PSA is applied to a surface under low pressure, it flows across the surface and forms close molecular contact with the surface contours (behaving similarly to a liquid) [90]. The substrate of a PSA is typically made from a soft material with a low Young’s modulus, enabling flexible and close contact with the surface. Given the viscoelastic properties of the PSA, PSA-based electrode patches can exert a strong adhesive force even on elastic skin with multi-scale roughness, under only slight pressure [91]. However, the use of PSA often results in skin injury and damage, and due to surface scaling and contamination, it is rarely reusable. To overcome these limitations, inspired by novel adhesion mechanisms observed in various animals, biomimetic adhesive electrode patches have been developed to replace PSA and meet the required specifications [92]. The biomimetic adhesive electrode patch comprises three components: nickel-based silver film microneedles, a biomimetic adhesive patch, and a metal connecting button. The nickel-based silver film microneedles are laser-cut into circular disks with a diameter of 10 mm, whilst the metal connecting button utilizes the fastening components found in conventional Ag/AgCl electrode patches [93].

Electrode patches are classified according to their signal acquisition methods into: wet electrode patches, dry electrode patches, and invasive electrode patches [94]. Wet electrode patches are simple to use and are the most commonly used type in modern medicine. Dry electrode patches can be used for extended periods and represent a key area of research for next-generation electrode patches. Invasive electrode patches bypass the skin’s surface barrier to extract bioelectric signals directly from within the body, thereby achieving greater accuracy in signal acquisition. As a primary focus of research into next-generation electrode patches, dry electrode patches have significantly expanded their applicability by utilizing dry adhesion technology to replace traditional chemical adhesives. Drawing inspiration from the crawling behavior of natural organisms such as large geckos and insects [95], the researchers have innovatively proposed a dry adhesion technology based on van der Waals forces. Kim et al. [96] developed a dry conductive electrode patch inspired by gecko setae using a PDMS-based conductive composite. By doping PDMS with a CNT mixture, they obtained an excellent conductive material, which was surface-modified with a microstructural array to provide adhesion. Not only does it adhere better to the skin, with an adhesion strength of approximately 1.3 N/cm^2^, but it also exhibits similar conductivity to traditional 3M electrodes and enables real-time ECG monitoring without the need for additional external devices. Furthermore, it provides adhesion strengths of 1.9 N/cm^2^ and 1.2 N/cm^2^ in dry and humid environments, respectively, with no significant impact on adhesion strength after repeated cycles.

**Figure 9:**
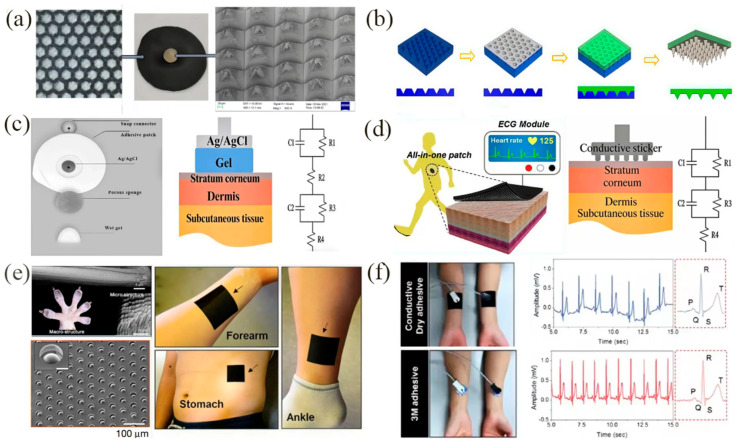
(**a**) Biomimetic adhesive electrode patch and its microstructure [97]. (**b**) Manufacturing process of nickel-based silver-coated microneedles [98]. Copyright 2025 OPEN ACCESS ACS. (**c**) Schematic diagram of the wet electrode patch and its bioelectrical signal acquisition [97]. Copyright 2025 OPEN ACCESS ACS. (**d**) Schematic diagram of the dry electrode patch and its bioelectric signal acquisition [97]. Copyright 2025 OPEN ACCESS ACS. (**e**) The van der Waals force-based adhesion mechanism of gecko setae, and the PDMS conductive composite electrode patch inspired by this. (**f**) Comparison of ECG waveforms acquired by the PDMS conductive composite electrode patch and conventional 3M electrodes [19]. Copyright 2023 OPEN ACCESS Aggregate.

Geckos and beetles possess structures consisting of nanometer- or micrometer-scale setae; although the van der Waals forces at individual contact points are weak, the vast number of micrometer-scale setae and nanometer-scale villi on the bodies of geckos and beetles ensures a sufficient number of contact points. The cumulative van der Waals forces generate sufficient adhesive force to support their own weight, enabling them to adhere firmly, reliably, repeatedly, and reversibly to surfaces with varying degrees of roughness and orientation [97]. Drawing inspiration from the adhesion mechanisms of geckos and beetles, densely packed microstructures have been designed on the surface of the conductive electrodes. These microstructures are tailored to closely match the scale of the skin’s surface, providing the necessary adhesion whilst also ensuring breathability (Figure 9a).

#### 3.4.3. Bionic Arrangement of Electrode Arrays

**Figure 10:**
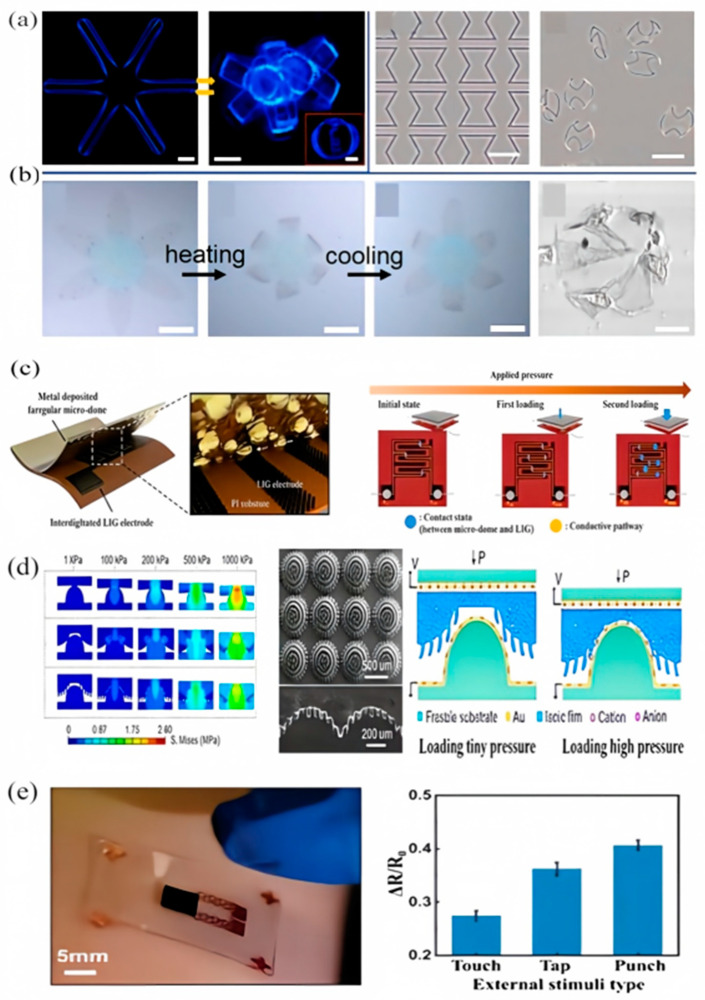
(**a**) Flower-shaped FLG/SU-8 bilayer in the flat and self-folded states. Scale bars are 200 μm [99]. (**b**) Optical microscope images of a flower-shaped thermally responsive graphene, which can reversibly fold and unfold [99]. (**c**) The sensing principle of the irregular microsphere structure [22]. Copyright 2022 OPEN ACCESS Advanced Functional Materials. (**d**) Schematic illustration of the operation of a flexible sensor utilizing the synergistic interaction between dome and microcolumn structures under different pressures [101]. Copyright 2022 OPEN ACCESS Advanced Functional Materials. (**e**) A composite microcolumn array patch adhered to the skin surface, generating a resistive signal in response to external mechanical stimulation [21]. Copyright 2024 OPEN ACCESS Advanced Functional Materials.

The arrangement of an electrode array directly influences the mechanical contact characteristics between the electrodes and the tissue, as well as the distribution of stress. Drawing inspiration from the natural arrangement of nerve bundles and blood vessels in the human body, a flexible, stretchable electrode array has been designed to ensure uniform contact between the electrodes and the tissue, thereby reducing localized stress concentrations. For neural electrodes, the parallel arrangement of nerve fibers is emulated by designing the electrode contacts as linear arrays with spacing controlled between 100 and 500 μm, matching the diameter of nerve bundles (100–1000 μm) (Figure 10a,b). Flexible interconnection structures, such as serpentine [99] and helical configurations [100], are employed. These structures effectively disperse strain and minimize stress concentration, thereby reducing the risk of mechanical failure during movement. Inspired by the art of paper cutting [98], these highly stretchable biosensors maintain high-fidelity electrophysiological signals even under extreme deformation (e.g., during heart or brain monitoring. Similarly, serpentine interconnections mitigate delamination in ECG sensors by transferring strain away from the active elements (Figure 9b). Furthermore, techniques such as metal buckling—where a metal film wrinkles over a pre-strained elastomer—enable stretchability without compromising electrical conductivity.

For skin electrodes mimicking the distributed arrangement of skin hair follicles, the electrode contacts were designed as spheres uniformly distributed across the substrate surface, with a contact diameter of 100–300 μm and a spacing of 500–1000 μm, ensuring close contact between the electrodes and the skin surface. Park et al. reported a method for generating irregular microsphere structures via the thermal expansion of imploding microspheres to fabricate sensors with microscale three-dimensional surface topography (Figure 9c). By utilizing composite films with varying microsphere concentrations, the microscale three-dimensional surface topography is controlled (Figure 10c). The hierarchical sensing resulting from the non-uniform contact variations between the irregular microsphere structure and the graphene electrodes endows the sensor with a sensitivity as high as (50.45 kPa^−1^) [22]. Guo et al. [101] combined microdome and micropillar structures, adding dense microscale pillars to the microdome structure to compensate for the gaps between them (Figure 10d). As pressure increased, the micropillars came into contact with the electrodes, gradually filling the gaps between the ion layer and the electrode structure, thereby reducing the frictional force between the structures.

The elastic microsphere structure deforms under external force and generates an elastic force to counteract the external pressure. When the elastic force is in equilibrium with the external force, the microsphere assumes a stable deformation and establishes a specific contact area. When subjected to different pressures, the microsphere undergoes varying degrees of deformation, resulting in different contact areas and different distances between the electrodes, thereby producing different electrical signals [102]. Although the stress concentration effect is relatively small due to the linear shape of the dome, the contact area varies considerably. At the same time, the unique shape of the sphere enables the detection and differentiation of mechanical stimuli of different directions and types. The combination of macrodomes and microcolumns, coupled with the collective behavior of the microcolumns, homogenizes the stress at the interface, thereby mitigating structural stiffening. Furthermore, the microcolumns minimize the initial contact area at the interface, thereby enhancing the sensitivity of the device [19]. To enhance the adhesion of the electrode patch to human skin, Bae et al. (Figure 10e), in [21], proposed a composite microcolumn array patch based on van der Waals forces. In this design, the shafts of the microcolumns are made of high-modulus PDMS, primarily to enhance the structural stability of the microcolumns, whilst the tips are made of low-modulus PDMS to improve conformability to rough skin surfaces and resistance to external mechanical stress, thereby ensuring stable electrical signals. However, most microdome structure electrodes exhibit poor linearity and are unable to maintain consistent sensitivity across the entire pressure range; this non-linear response means that pressure sensors require additional signal processing to obtain accurate pressure readings. Furthermore, microdome structures have limited deformation and low compressibility, making them prone to saturation under external forces, which severely limits their sensitivity under high-pressure conditions [103]. Mechanical biomimicry reduces the mechanical disparity between the biomimetic electrode and biological tissue, minimizing pressure and irritation on soft tissues at the interface and thereby reducing tissue damage. This endows the electrodes with excellent flexibility and extensibility, enabling them to adaptively deform in response to tissue movement. They maintain close contact with the interface under dynamic conditions such as bending and stretching, ensuring stable electrical conduction pathways. This prevents interruptions or attenuation in signal acquisition and electrical stimulation, thereby significantly enhancing the electrodes’ operational stability and long-term reliability in physiological environments.

### 3.5. Functional Bionics

**Figure 11:**
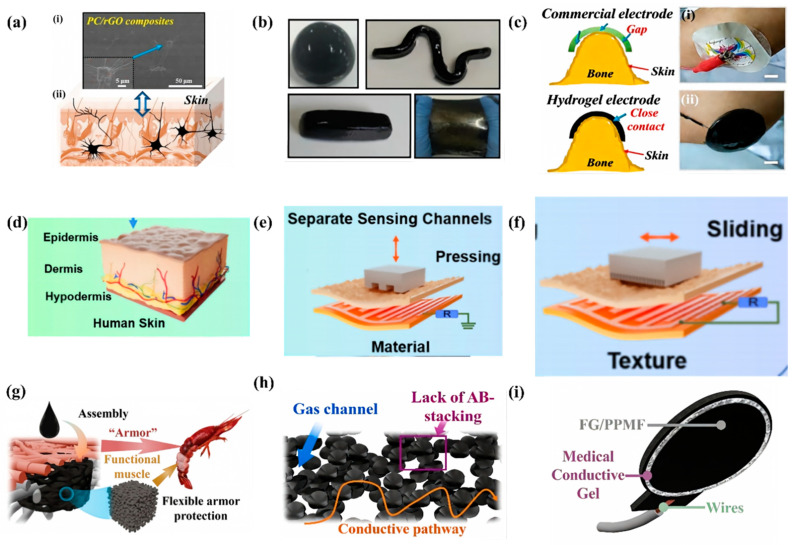
**Functional hydrogel.** (**a**) (i) SEM image of the neural-like PC/rGO network, (ii) 3D image of tactile nerve distribution in human skin [9]. Copyright 2019 Chemical Engineering Journal. (**b**) PC/rGO/PVA hydrogel can be tailored into various shapes (spherical, curved, and cuboid) and even stretched into a skin-like film [9]. Copyright 2019 Chemical Engineering Journal. (**c**) Schematic illustration of conformal contact between commercial electrodes and hydrogel electrodes, along with digital photographs of conformal contact for (i) commercial electrodes and (ii) PrP hydrogel electrodes (scale bar: 1 cm) [9]. Copyright 2019 Chemical Engineering Journal. (**d**) Schematic illustration depicting the detailed structure [11]. Copyright 2023 ACS Nano. (**e**) Sensing mode [11]. Copyright 2023 ACS Nano. (**f**) Application scenarios of BHES as a human−machine interaction interface [11]. Copyright 2023 ACS Nano. (**g**) “Armor” design strategy for the FG/PPMF electrode, where PPMF acts as a protective layer for the internal FG components, forming a porous conductive network. This resembles a crayfish shell protecting the crayfish muscle. The turbostratic characteristics of FG facilitate the formation of this porous structure. FG, in the form of an ink, is assembled in situ deep within the PPMF to create FG/PPMF [13]. Copyright 2024 Advanced Science. (**h**) Schematic diagram of the breathability mechanism of the FG/PPMF electrode, attributable to the turbostratic arrangement of FG [13]. Copyright 2024 Advanced Science. (**i**) FG/PPMF electrode patch for monitoring electrophysiological signals [13]. Copyright 2024 Advanced Science.

The core functional simulation of biomimetic ECG electrodes involves abstracting, decomposing, and engineering key skin functions, addressing multiple failure modes of the skin–electrode interface under real monitoring conditions (Figure 11a), essentially an application-driven interface engineering issue. Current research has advanced from early single-point efforts to replace Ag/AgCl electrodes to systematic, modular functional bionic systems. Functionally, existing biomimetic ECG electrodes comprise three core modules: (1) A bio-inspired electrical coupling and conductive stability module, mimicking cutaneous neural network connectivity via interconnected, reconfigurable conductive networks to maintain electrical continuity during deformation, suppress microcracks and resistance fluctuations, and ensure high-quality ECG signal acquisition. (2) A bio-inspired mechanical compliance and anti-motion artifact module, replicating the layered mechanical division of the skin’s epidermis and dermis through layered or strain-isolation designs to mitigate motion-induced electrical coupling disruption. (3) A bio-inspired skin microenvironment and long-term stability module, mimicking skin’s perspiration and moisture regulation via breathable/porous structures to alleviate signal drift from sweat accumulation and heat buildup. Supporting modules (e.g., conformal contact, reversible adhesion, biocompatibility) further affect user experience and system reliability. The development trend shifts from single-module optimization to multi-module synergy, prioritizing scenario-tailored overall performance over individual metric maximization. This evolution reflects progress from imitating single skin physical properties to systematic reconstruction of the skin–electrode interface’s overall functional behavior, laying a critical foundation for extending wearable ECG monitoring from short-term, controlled settings to long-term, real-life applications.

#### 3.5.1. Neural-like Conductive Network Hydrogel Electrodes

Neural-like Conductive Network Hydrogel Electrodes are a type of dry electrode inspired by the biomimetic prototype of the “high connectivity and signal continuity under deformation” characteristic of nerve fiber networks in natural skin [9] (Figure 11b). The core of this work is not merely conductive filler incorporation, but the pursuit of “network topological stability” to sustain unbroken conductive pathways under stretching, bending, and compression. In biomimetic design, these electrodes mainly realize structural and material biomimicry: structurally, an interconnected, reconfigurable conductive nanonetwork mimics neural network connectivity, enabling conductive pathways to maintain electrical continuity via network rearrangement/sliding/re-contacting upon deformation, thus reducing resistance fluctuations and noise from microcracks and local disconnections. Materially, the high-water-content hydrogel (ionic conductor/flexible matrix) acts as a “tissue-like interfacial layer,” promoting stable ionic–electronic coupling with the stratum corneum. Additionally, interfacial biomimicry (tissue-like hydrogel properties) reduces interfacial impedance and enhances electrical coupling with the skin, while mechanical biomimicry (hydrogel-imparted high ductility and conformability) ensures conformal contact on high-curvature areas (e.g., elbow, clavicle). Process-wise, its simple fabrication enables large-area, thin-film preparation, yielding skin-attachable, stretchable macro-morphology. Key challenges include ensuring conductive network uniformity/repeatability and hydrogel hydration stability. For performance evaluation: electrically, low interfacial impedance and controlled resistance variation under deformation are critical (especially for ECG, as baseline drift and low-frequency noise impair P-QRS-T wave recognition); mechanically, high ductility and conformability are featured; and biocompatibly, the hydrogel matrix is skin-friendly with low irritation, but prolonged wear may cause dehydration, increasing impedance, stiffening mechanics, reducing adhesion, and degrading signal stability. In ECG applications, these electrodes excel in high-curvature areas, mild dynamic conditions, and short-to-medium-term wear (e.g., clear waveforms at the elbow/clavicle, suppressed noise from conductive disconnections during motion), though motion artifacts may increase without additional mechanical constraints under vigorous movement-induced interfacial shear. Mechanistically, the conductive nanonetwork’s topological stability enables dynamic pathway adjustment (rearrangement/sliding/re-contacting) upon deformation, avoiding abrupt resistance changes; meanwhile, the hydrogel’s high-water content provides ionic conductivity and a favorable ion–electron coupling interface with the stratum corneum, laying the material foundation for stable ECG signal acquisition.

#### 3.5.2. Elastomer-Conductive Hydrogel Layered Integrated Electrodes

The biomimetic prototype for elastomer-conductive hydrogel layered integrated electrodes is more explicitly rooted in the “layered mechanical division of labor between the skin’s epidermis and dermis (Figure 11b).” [11]. Their functional simulation targets motion artifacts—the key challenge in dynamic scenarios—defined as electrode–skin micro-slippage under shear and localized strain, which induces contact impedance fluctuations and low-frequency drift. In biomimetic design, these electrodes primarily adopt structural and mechanical biomimicry. Structurally, a bilayer system is constructed: a high-modulus/high-toughness elastomeric support layer (epidermis-mimicking) and a low-modulus ionic conductive hydrogel coupling layer (dermis-mimicking) (Figure 11c), which distributes skin motion-induced deformations. Mechanically, the elastomer layer provides stabilization and tear resistance to restrict hydrogel overstretching, while the hydrogel layer enables low-impedance skin coupling and buffers microscopic irregularities, reducing interfacial shear and slippage (Figure 11d). Interfacial and material biomimicry are also embodied: interfacial reliability is enhanced via in situ curing, interpenetrating networks, or chemical/physical bonding to avoid delamination under repeated deformation and sweating; the elastomer improves processability and structural support, facilitating stable fabrication and repeatable wear. Performance evaluation focuses on three aspects: electrically, low static impedance, minimal dynamic impedance fluctuations, stable ECG baselines, and reduced motion artifacts; mechanically, a balance between conformability and resistance to tear/fatigue; and biocompatibly, skin safety of both phases and no irritating residues from interlayer adhesion. This electrode is ideally suited for dynamic ECG monitoring (Figure 11e), maintaining clear P-QRS-T structures with minimal baseline drift during wrist motion, fist clenching, or walking. The support layer enables practical product forms (patches, wristbands), advancing engineering implementation for real-life ECG monitoring. Mechanistically, layered mechanical division of labor suppresses interfacial shear: the high-modulus elastomer absorbs tensile/shear stresses to protect the hydrogel, while the low-modulus hydrogel conforms to skin microtopography for stable contact, converting destructive shear into controlled elastic deformation and reducing impedance fluctuations (Figure 11f). However, it presents engineering trade-offs: complex structure, narrow process window, strict requirements for long-term interfacial reliability and batch consistency, and potential hydrogel dehydration. Long-term stability thus requires synergy with microenvironment regulation or non-hydrogel strategies.

#### 3.5.3. Biomimetic Skin-Breathing Gas-Permeable Electrodes

Biomimetic skin-breathing gas-permeable electrodes draw their biomimetic prototype from a frequently overlooked but critically important function of the skin as an organ for long-term wear: “respiration and moisture vapor exchange (microenvironmental homeostasis).” [13]. In many failed cases of wearable ECG monitoring, the problem does not arise within the first 10 min but after several hours: sweat accumulation and heat buildup lead to stratum corneum hydration swelling and changes in the interfacial state, resulting in degraded adhesion, impedance fluctuations, and increased noise, which in turn cause ECG baseline drift and misidentification of characteristic waves (Figure 11g). In terms of biomimetic design, these electrodes primarily embody structural and interfacial biomimicry: Structurally, they employ porous/permeable structures to enable continuous moisture vapor and gas exchange, thereby reducing interfacial moisture accumulation and temperature rise, effectively transforming “time-degraded interfacial drift” into “a more stable long-term interfacial state.” Interfacial biomimicry is manifested in the porous structure achieving a compromise between contact area and breathability, avoiding complete closure that causes skin maceration while maintaining sufficient effective contact points and ion–electron coupling pathways. Furthermore, these electrodes also embody material and mechanical biomimicry. Material biomimicry is reflected in the construction of stable and repeatable pore structures and conductive networks (e.g., microporous elastomers, nonporous conductive layers, mesh/armor-like geometries), ensuring that the pore structures do not collapse, crack, or lose electrical connectivity after repeated bending, stretching, sweat infiltration, and friction/cleaning. Mechanical biomimicry requires geometric and material design to counteract the stress concentration introduced by the pore structures, ensuring fatigue life and structural stability during attachment/detachment cycles. In terms of performance evaluation, electrically, in addition to initial impedance, greater emphasis is placed on a flatter impedance variation curve during long-term wear and noise that does not significantly increase over time. Mechanically, the focus is on the impact of the pore structure on fatigue life. Regarding biocompatibility, advantages are often more readily demonstrated: improved breathability leads to lower skin irritation risk, better comfort, and fewer instances of erythema/maceration (though material safety verification remains necessary). In terms of ECG application scenarios, the most compelling use cases for this electrode type are typically long-term continuous ECG monitoring (e.g., 24 h monitoring, sleep monitoring, remote follow-up). The value of “breathable biomimicry” is particularly evident in sweaty or high-temperature environments: its purpose is not to make the ECG momentarily cleaner, but to ensure it remains reliable and usable over timescales of hours. From a mechanistic perspective, the core principle lies in reconstructing the skin–electrode interfacial microenvironment through porous structures to achieve continuous moisture vapor and gas exchange. When sweat is produced, the porous channels allow for vapor evaporation or drainage, preventing sweat accumulation and the formation of a liquid film at the interface (Figure 11h). This, in turn, prevents stratum corneum hydration swelling, adhesion degradation, and impedance fluctuations. Simultaneously, a stable conductive network ensures the continuity of electrical pathways. This design effectively transforms “time-degraded interfacial drift” into “a more stable long-term interfacial state.” However, its limitation lies in the inherent trade-off between high breathability, high conductivity, and strong adhesion. Excessively high porosity may compromise effective contact area and the advantages of low impedance. Therefore, synergistic design with conductive network biomimicry (such as the connectivity strategy of neural-like networks) or mechanical layering biomimicry (such as the constraint strategy of elastomer–hydrogel systems) is necessary to simultaneously meet the dual requirements of dynamic stability and long-term reliability.

Looking at the overall research progress, “functional biomimicry” in biomimetic ECG electrodes has formed a relatively clear and continuously evolving modular system. The research focus is no longer confined to single materials or individual performance metrics. Instead, it revolves around abstracting, decomposing, and engineering the key functions of the skin, specifically targeting the multiple failure modes of the skin–electrode interface under real-world ECG monitoring conditions. Unlike the earlier approach of simply “replacing Ag/AgCl electrodes,” the current functional biomimicry in this field places greater emphasis on the stability of the interfacial system across three dimensions: time, motion, and environmental disturbances. Its essence is an interface engineering problem driven by application scenarios. From a functional perspective, the biomimetic modules in existing biomimetic ECG electrodes can be broadly categorized into several interrelated directions with distinct emphases. The first is the bio-inspired module for electrical coupling and conductive stability. This module focuses on maintaining stable electrical connections and low interfacial impedance under conditions of skin deformation (Figure 11i). Its biomimetic objects are often derived from the characteristics of the cutaneous neural network or continuous conductive pathways within tissues. Through design strategies such as continuously interconnected conductive networks, synergistic ion–electron conduction, and compliant matrices, it reduces abrupt resistance changes and noise amplification caused by microcracks, local disconnections, or variations in contact area. This module provides the most fundamental electrical guarantee for high-quality ECG signal acquisition and is particularly decisive for low-frequency baseline stability in ECG recordings. The second is the bio-inspired module for mechanical compliance and anti-motion artifact functionality, which addresses motion artifacts—a core issue that has long constrained wearable ECG monitoring. Natural skin is not simply a soft medium but possesses a layered structure and stress distribution capabilities. Therefore, numerous studies have begun drawing inspiration from the mechanical division of labor between the skin’s epidermis and dermis. Through layered structures, strain isolation, or mechanical constraint designs, they transform complex human motions into more controllable interfacial deformations. The value of this functional biomimetic module lies not in “completely eliminating motion,” but in suppressing the disruption of electrical coupling caused by interfacial shear and micro-slippage, enabling the ECG electrode to maintain an acceptable signal-to-noise ratio and waveform stability under dynamic conditions. The third important direction is the bio-inspired module for skin microenvironment and long-term stability. Its biomimetic objects are no longer limited to the mechanical or electrical properties of the skin but extend to the skin’s organ-level functions of respiration, heat dissipation, and moisture vapor regulation. Extensive practical experience has shown that many biomimetic ECG electrodes exhibit excellent performance in the short term, but after prolonged wear, they suffer from impedance drift and signal degradation due to sweat accumulation, heat buildup, skin maceration, or material aging. Therefore, reconstructing the skin–electrode interfacial microenvironment through breathable, porous, permeable structures, or novel stable ionic conduction systems has become an indispensable component of functional biomimicry. This module directly determines whether biomimetic ECG electrodes can transition from laboratory demonstrations to all-weather, real-life scenarios. Furthermore, beyond these core modules, there are several supporting functional biomimetic modules. These include, for example, biomimicry of epidermal microtextured surfaces for conformal contact (enhancing the effective contact area), biomimicry of adhesion and reversible attachment (maintaining long-term adhesion while reducing skin damage), biomimicry of biocompatibility and skin-friendliness (minimizing irritation and inflammation risk), and biomimicry of reusability and maintainability (serving practical wear and productization needs). These modules often do not individually determine ECG signal quality, but they significantly influence user experience and system reliability in real-world applications. From a development trend perspective, functional biomimicry in ECG electrodes is undergoing a clear transition from “single-module optimization” to “multi-module synergy.” Early research often focused on achieving peak performance in a single function, such as the lowest impedance or the highest stretchability. However, an increasing number of current studies are recognizing the inherent coupling and trade-offs between different biomimetic functions—for example, the balance between conductive continuity and breathability, softness and structural stability, or strong adhesion and skin comfort. Therefore, future, more competitive biomimetic ECG electrode designs will likely not achieve extreme values in a single metric, but rather achieve optimal overall performance through the rational combination of multiple functional biomimetic modules tailored to specific ECG application scenarios. In summary, the functional biomimetic modules in biomimetic ECG electrodes have evolved from imitating a single physical property of the skin to achieving a systematic reconstruction of the overall functional behavior of the skin–electrode interface. This shift has not only promoted the stable acquisition of ECG signals under complex conditions but has also laid a critical technological foundation for expanding wearable ECG monitoring from short-term, controlled environments to long-term, real-life settings. In the future, with the further integration of materials science, structural design, and manufacturing processes, functional biomimetic modules will be more explicitly tailored to serve specific ECG application scenarios, becoming an important bridge connecting fundamental research and practical application.

Taking into account the mechanisms of action, typical biomimetic prototypes and the optimization results for electrodes across the five key dimensions of biomimetic design—structure, interface, materials, mechanics and function—it is evident that different biomimetic design approaches have their own specific focuses when optimizing the performance of ECG electrodes, whilst also facing inherent limitations in terms of manufacturing processes, performance, and suitability for specific applications. A comparison of representative biomimetic solutions is shown in Table 3:

### 3.6. Micro-Nano Fabrication Processes of Biomimetic ECG Electrodes

#### 3.6.1. Soft Lithography and Master Mold Replication (Standardized Fabrication for Structural Biomimicry)

Soft lithography relies on SU-8 photoresist to fabricate rigid silicon master molds. Elastomers, including PDMS and silk fibroin, are cast, cured, and demolded to replicate three-dimensional biomimetic microstructures with a high precision of ±1 μm. Sequential multilayer casting enables the construction of gradient-modulus composite substrates mimicking the epidermal–dermal bilayer of human skin, which achieves adhesive contact with skin without adhesive glue via microscale van der Waals forces. This technique constitutes the standard laboratory fabrication route for gecko-seta-inspired and sponge-porous dry electrodes. Biomimetic dry electrodes fabricated by Kim’s group using gradient micropillar master molds drastically suppress motion artifacts in dynamic ECG monitoring. Porous PD electrodes prepared by Lo’s group via sponge template casting can store trace conductive gel within internal pores, yielding merely 12% impedance drift over 72 h of continuous recording. Nevertheless, this process suffers from inherent limitations for mass production: high cost of silicon master molds, frequent microstructure damage during large-area demolding, and incapability of forming continuous nanofiber networks [1,2].

#### 3.6.2. Electrospinning and Melt Electro Writing (MEW) Fiber Biomimetic Fabrication (Dedicated for Breathable Epidermal and Implantable Electrodes)

Electrospinning generates nanofibers with diameters ranging from 50 to 1000 nm under a high-voltage electric field. Coaxial electrospinning facilitates the construction of hydrophobic–hydrophilic Janus bilayer architectures for unidirectional sweat drainage. Melt electro writing (MEW) fabricates gradient-modulus fibrous scaffolds by stacking molten microfilaments. The combination of the two techniques is tailored separately for breathable epidermal electrodes worn on skin and myocardial implantable electrodes. Three-layer electrospun biomimetic cactus films reported by Chen’s group exhibit a water vapor transmission rate of 3795 g·m^−2^·d^−1^, trigger no skin irritation after 7 consecutive days of wearing, and deliver an ECG SNR 58 times higher than commercial gel electrodes. MEW-fabricated stratified myocardial fibrous scaffolds possess moduli matching native cardiac tissues and induce no fibrotic inflammation for four weeks post-implantation. However, this process is constrained by frequent filament breakage upon conductive filler doping, poor precision in fiber orientation control, and inability to fabricate micrometer-scale protrusive adhesive structures [3,4].

#### 3.6.3. Direct Ink Writing (DIW) Additive Manufacturing for Integrated Multidimensional Biomimetic Forming

Direct ink writing (DIW) 3D printing adopts PEDOT:PSS composite conductive hydrogel as extrusion ink and eliminates the requirement for prefabricated master molds. Biomimetic topologies such as shrimp shell armor, serpentine strain–relief structures, and bifurcated neural networks can be deposited layer-by-layer at room temperature. Tunable printing density enables in situ formation of gradient mechanical stratification, simultaneously generating breathable pores and continuous conductive pathways. Composite electrodes integrating elastic supporting layers and ionic coupling layers can be fabricated in a one-step workflow. Biphasic micropatterned electrodes printed via this process sustain 100% tensile strain without conductive pathway disconnection, and ECG motion artifacts are reduced by 71% under strenuous exercise conditions. The main drawback lies in its minimum forming resolution of only 20 μm, which restricts the preparation of nanoscale refined biomimetic architectures. Additionally, continuous roll-to-roll mass production efficiency is low, rendering the technique only applicable to small-batch laboratory customization [5].

#### 3.6.4. Printed Electronics (Core Technology for Industrial Mass Production)

Printed electronics integrate three complementary processing workflows: screen printing, inkjet modification, and roll-to-roll blade coating. Screen printing enables batch fabrication of electrode arrays; inkjet printing realizes localized conductive modification of microstructures; and roll coating continuously produces large-area flexible conductive films. This technique is commonly cascaded with semi-finished products from electrospinning and soft lithography for mass manufacturing and remains the sole low-cost manufacturing route compatible with standardized medical device production lines to date. Liquid metal composite epidermal electrodes fabricated via roll-to-roll coating display negligible ECG baseline drift over 24 h under profuse sweating, with single-batch output reaching thousands of devices and drastically reduced manufacturing costs. A standalone printing process can only form two-dimensional planar thin films and cannot independently construct 3D biomimetic micro protrusions, thus requiring pre-positive structural forming procedures as a prerequisite [6].

#### 3.6.5. Laser Microfabrication and Laser-Induced Graphene (LIG) Processes for Ultrathin Epidermal Electrodes

Laser fabrication branches into two technical routes: femtosecond laser ablation and laser-induced graphene (LIG). Femtosecond laser patterning constructs serpentine strain-dissipating interconnects to disperse deformation stress. The LIG technique directly carbonizes polyimide (PI) substrates in situ to generate porous graphene conductive networks, realizing seamless integration of substrate and conductive layers in a single step. Ultrathin electrodes fabricated by combining LIG with silver nanowire transfer printing feature an ultra-low modulus of 0.53 MPa, conform tightly to high-curvature skin regions including wrists and clavicles, and output complete, distinct ECG waveforms under dynamic scenarios such as running and swimming, with superior SNR compared to commercial 3M gel electrodes. Limitations include narrow substrate compatibility of the process, incapability of processing water-containing hydrogel biomimetic materials, and challenges in precise regulation of carbonization uniformity over large areas [7].

#### 3.6.6. Sacrificial Layer Transfer Printing and Heterogeneous Interface Composite Fabrication (Biomimetic Anti-Damage Interface Preparation)

This process adopts water-soluble PVA thin films as temporary sacrificial carriers. MXene and noble metal nanoconductive films are first deposited on rigid substrates, then fully transferred onto flexible biomimetic substrates to seamlessly construct mussel-inspired multistage porous, amphiphilic wetting ionic–electronic coupling interfaces, which effectively mitigate interfacial delamination between rigid and soft material layers. RGD peptide modification can be introduced during transfer printing to enhance the cytocompatibility of implantable electrodes. Mussel-inspired ionic skin fabricated via solid-phase self-assembly combined with sacrificial transfer printing maintains stable ECG signal conduction even with surface scratches and localized fractures, showing no obvious signal attenuation during underwater monitoring. However, the entire multi-step wet preparation workflow is cumbersome; dissolution of sacrificial layers tends to damage the porous structures of hydrogels, leading to low yield in mass production [8].

#### 3.6.7. Standardized Composite Manufacturing Routes Integrating Multiple Processes

A single micro-nano fabrication process can only optimize one biomimetic performance metric. High-performance long-term ECG electrodes must simultaneously satisfy four core requirements: three-dimensional adhesive structures, breathable sweat drainage, gradient mechanical matching, and uninterrupted conductive pathways. Three mature integrated manufacturing schemes have been established: wearable patch route for consumer electronics: soft lithography to fabricate gecko-inspired microadhesive substrates, combined with roll-to-roll conductive hydrogel coating and laser array cutting [1,6,7]; high-performance breathable epidermal electrodes: electrospun Janus fiber skeletons integrated with DIW-printed conductive networks and inkjet adhesive modification [3,5]; and clinical implantable electrodes: MEW gradient fibrous substrates coupled with LIG conductive layers and sacrificial-layer peptide interface modification [4,7,8].

Micro-nano fabrication acts as the core engineering bridge connecting biomimetic design theories and tangible ECG electrodes. The six aforementioned processes possess distinct functional positioning: soft lithography serves as the primary candidate for fabricating micrometer-scale 3D adhesive microstructures [1,2]; electrospinning and MEW target breathable epidermal and fibrous implantable biomimetic devices [3,4]; DIW additive manufacturing is tailored for the development of integrated multidimensional composite electrodes [5]; printed electronics constitutes the mainstream mass-production technology for consumer wearable products [6]; the LIG laser process specializes in ultrathin epidermal electrodes adapted to high-curvature skin [7]; and sacrificial layer transfer printing is deployed for anti-damage implantable devices with biomimetic interfaces [8]. OMIEC material processing supplies systematic theoretical underpinnings for ionic–electronic coupling of electrode interfaces [9]. Future research should center on three pivotal directions: integrated multi-process composite manufacturing systems, bio-based green fabrication, and digital continuous production lines, to balance processing precision, manufacturing cost, and long-term interfacial stability. Such advances will facilitate the widespread application of biomimetic ECG electrodes across clinical monitoring, home-based chronic disease screening, and sports health management.

### 3.7. Fundamental Mechanisms of Bioelectrical Stimulation and In Vivo Current Distribution

Human skin consists of three electrically heterogeneous layers—the stratum corneum, epidermis, and dermis—each exhibiting vastly different electrical conductivities. All cutaneous bioelectrical monitoring systems operate under low-frequency quasi-static Maxwell’s equations. At low frequencies, displacement current is negligible, leaving conductive current within tissues as the sole carrier of physiological signals. Mismatch between electronic and ionic charge carriers at the electrode–skin contact interface induces the formation of an electrical double layer (EDL). The resulting interfacial impedance, composed of charge-transfer resistance and double-layer capacitance, constitutes the primary origin of motion artifacts, baseline drift, and attenuated deep-tissue physiological signals.

Two dominant current excitation modalities are widely adopted in contemporary biosensing, which generate fundamentally distinct three-dimensional in vivo current distributions.

Monopolar adjacent potential (MAP) excitation current propagates laterally across the superficial epidermis exclusively between paired electrodes, with current density highly concentrated at the skin surface and rapid attenuation in subcutaneous tissues. This modality yields higher amplitude superficial voltage signals yet suffers from spatially limited sensitivity restricted to shallow cutaneous layers. Conductive abnormalities in subcutaneous tissues are readily masked, rendering MAP only suitable for short-term, localized superficial monitoring.

Monopolar opposite potential (MOP) excitation, with widely spaced excitation electrodes, enables vertical electric field penetration through the full stratified skin structure, yielding spatially uniform three-dimensional current distribution across tissue volumes. MOP delivers statistically significant improvements (*p* < 0.001) in three core performance metrics: full-domain impedance contrast, lesion localization error, and system SNR. It is therefore tailored for long-term wearable devices and continuous acquisition of subcutaneous physiological signals. Interfacial impedance directly modulates global in vivo current propagation pathways. When interfacial impedance exceeds 10 kΩ·cm^2^, the majority of applied excitation voltage dissipates across the skin surface, drastically attenuating effective current injected into subcutaneous layers and eliminating detectable signals from deep tissue regions. When interfacial impedance is stably confined to the range of 1–10 kΩ·cm^2^, fluctuations in characteristic detection indices (ΔZ, SNR) induced by impedance variation remain below 5%, ensuring robust and reproducible comparative analysis of system performance. Biomimetic microstructures inspired by gecko setae, octopus suction cups, and porous sponges deliver core electrical advantages: expanding the effective electrode contact area, dispersing localized interfacial current density to mitigate interfacial polarization, and maintaining consistent skin–electrode contact during limb deformation, thereby preventing drastic distortion of in vivo current profiles induced by body movement.

### 3.8. Synergistic Design of Sensors, Circuits and Algorithms

#### 3.8.1. Hardware Matching Design of Biomimetic Electrodes and Analog Front-End (AFE) Circuits

Biomimetic dry electrodes and hydrogel electrodes exhibit high interfacial impedance ranging from kiloohms to megaohms, which readily induces baseline drift in conventional readout circuits; therefore, customized hardware architectures are required to accommodate their electrical characteristics [9]. A chopper-stabilized instrumentation amplifier is adopted in the proposed system, delivering an input impedance ≥ 1 GΩ and bias current < 200 fA. Combined with a right-leg drive circuit, the common-mode rejection ratio (CMRR) reaches 130 dB, enabling effective suppression of 50 Hz power-line interference. After monolithically integrating the ADS1299 ECG-specific chip with alginate PSTP biomimetic hydrogel electrodes, the ECG baseline drift under motion is reduced by 82%, accompanied by remarkable improvement in the integrity of QRS complexes [8]. Flexible printed circuit (FPC) boards and anisotropic conductive films are utilized to realize monolithic integration of electrodes and circuitry. Serpentine and spiral flexible interconnects are deployed to accommodate skin deformation and eliminate frictional noise. The fabricated tattoo-like flexible patch integrates porous biomimetic electrodes, filter components, and an on-chip microcontroller unit (MCU), with an overall thickness below 100 μm and a steady SNR ≥ 32 dB during dynamic monitoring [11]. Targeting the noise signatures of biomimetic electrodes, a hardware circuit combining a 0.05–40 Hz bandpass filter and a 50 Hz notch filter is configured. This architecture effectively suppresses sweat-induced low-frequency drift and reduces the computational overhead of post-processing algorithms, catering to long-term wearable monitoring with porous biomimetic electrodes.

#### 3.8.2. Coordinated Noise Reduction via Hardware–Embedded Algorithms for Motion Artifact Suppression

Microsliding of electrodes, sweat fluctuations, and fracture of conductive networks constitute the primary sources of motion artifacts. Structural optimization of electrodes alone cannot completely eliminate baseline distortion, necessitating synergistic noise suppression through hardware sensing and embedded algorithms. A three-axis accelerometer is integrated into the system to synchronously capture motion data. The embedded algorithm adaptively adjusts wavelet filter coefficients according to motion intensity: an extended filtering window is activated during strenuous exercise to suppress baseline drift, while narrowband filtering is applied under resting conditions to preserve subtle ST-segment features. Characterizations conducted on octopus-sucker-inspired multi-stage biomimetic electrodes demonstrate distortion-free ECG waveforms under dynamic motion, a 91% improvement in arrhythmia recognition accuracy, and a 16 dB elevation in ECG SNR [10]. A dynamic impedance detection branch is added to the hardware for real-time tracking of interfacial impedance variations. For abrupt impedance surges triggered by sweating or degraded electrode–skin contact, artificial intelligence (AI) algorithms perform baseline correction and waveform reconstruction, supporting 7 consecutive days of stable continuous recording with alginate- and sponge-based biomimetic electrodes. To address localized scratches and open-circuit faults of mussel-inspired damage-resistant biomimetic electrodes, multi-channel hardware-redundant acquisition is combined with an interpolation reconstruction algorithm. Missing signals are complemented using data from adjacent leads, maximizing the damage-tolerant monitoring performance of electrodes.

#### 3.8.3. Architecture of an Integrated Multimodal Intelligent Monitoring System

A five-dimensional biomimetic electrode is integrated with signal acquisition, wireless transmission, and edge computing modules to construct an all-in-one multimodal ECG monitoring system, breaking the limitations of conventional single-parameter monitoring [12]. A dedicated ECG system-on-chip (SoC) is employed, which integrates amplifiers, analog-to-digital converters (ADCs), and Bluetooth transceivers. Programmable gain is embedded to adaptively match the impedance of diverse biomimetic electrodes. The entire device measures 65 × 52 × 12 mm and achieves 72 h of continuous monitoring on a single charge [8,9]. ECG electrodes and a temperature-humidity sensor array are co-fabricated on a unified flexible substrate. Time-division multiplexing of circuits enables synchronous acquisition of multiple physiological signals. Integrated edge AI algorithms predict impedance drift based on skin humidity readings and trigger adaptive noise reduction in advance, rendering the system suitable for high-intensity exercise with profuse sweating. Flexible triboelectric nanogenerator (TENG) circuits are combined with graded-modulus gecko-inspired biomimetic electrodes to harvest mechanical energy from human body motion for self-powering. Low-power signal conditioning circuits and lightweight filtering algorithms are further incorporated to realize battery-free, long-duration ECG monitoring without reliance on lithium batteries.

## 4. Application Scenarios

### 4.1. Structural Biomimetics

**Figure 12:**
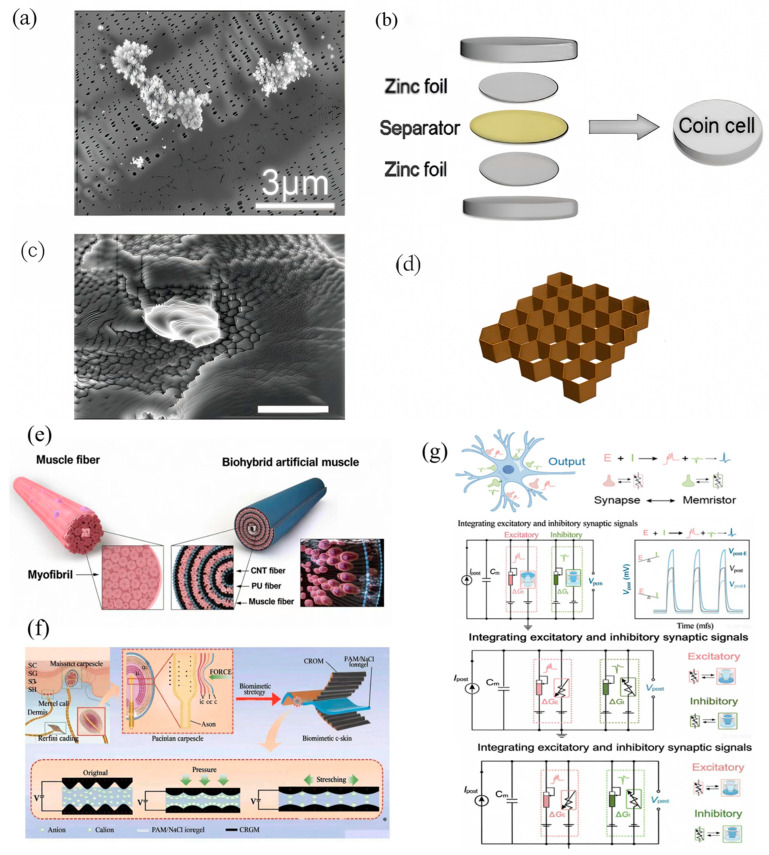
**The schematics show the structural biomimetics applied in the new-type electrodes:** (**a**) SEM image of zinc dendrites penetrating through a Celgard 2400 separator [104]. Copyright 2019 ACS Nano. (**b**) Configuration of the symmetrical Zn/electrolyte/Zn cell [104]. Copyright 2019 ACS Nano. (**c**) Zinc foil surface after cycling with PEO separator [104]. Copyright 2019 ACS Nano. (**d**) The preparation process of ALBC nanocomposite [29]. Copyright 2024 Advanced Functional Materials. (**e**) Similar to muscle fibers, the biohybrid artificial muscle is composed of carbon nanotube fibers, PU fibers, and skeletal muscle fibers [52]. Copyright 2021 OPEN ACCESS Nature. (**f**) Biomimetic strategy for human skin mechanoreceptors, and the ionized capacitance sensing mechanism of the biomimetic electronic skin under pressure and stretching [107]. Copyright 2024 OPEN ACCESS ACS. (**g**) Ideas for functional biomimetics of synapses and partial equivalent circuits [110]. Copyright 2025 OPEN ACCESS ACS.

Structural biomimetics focuses on imitating the multi-scale porous structures and highly ordered geometric arrangements of organisms to optimize the effective contact area of ECG electrodes, shorten the electrical signal transmission path, and improve the sensitivity and stability of ECG signal acquisition. In the fields of clinical ECG monitoring and wearable ECG devices, the microstructural design of electrodes plays a decisive role in the quality of ECG signal acquisition. Drawing on the micro-nano hierarchical biomimetic structure constructed on the surface of lotus leaves in nature and the multi-level pore system formed by imitating aerogels, the performance of ECG electrodes can be significantly optimized from the aspects of interface contact and signal conduction. Such biomimetic structures can effectively increase the specific surface area of the electrode, greatly increase the number of contact sites between the electrode and the skin, and at the same time enhance the sufficient wetting of the electrode bulk by the electrolyte gel, shorten the electrical signal transmission path, accelerate the signal conduction rate, thereby reducing the contact impedance, significantly improving the clarity of ECG signal acquisition, effectively suppressing interference signals, and ensuring the stability of long-term monitoring (Figure 12a) [104]. In dynamic ECG monitoring scenarios, especially for real-time ECG acquisition during exercise, ECG electrodes with coral-like or sponge-like porous structures show outstanding advantages. Such biomimetic morphologies have both high specific surface area and continuously connected transmission channels, which not only provide sufficient contact sites for electrical signal acquisition but also efficiently realize the uniform distribution of electrolytes and the timely discharge of metabolites, reduce the impact of skin deformation on electrode contact during exercise, avoid signal drift, and thus significantly improve the accuracy, stability, and durability of dynamic ECG monitoring [105]. For example, the research by Li-Wei Lo et al. demonstrated a soft sponge electrode that can be manufactured at low cost and scaled up, which is very suitable for long-term and motion artifact-resistant high-precision biopotential signal recording. The sponge electrode has a simple structure, including a porous PDMS sponge covered with a conductive PEDOT:PSS layer. There are hundreds of micrometers of micropores inside the sponge, which greatly increases the effective contact area between the skin and the electrode, leading to a significant decrease in skin–electrode contact impedance and an increase in SNR. In addition, compared with traditional planar electrodes, the porous structure of the sponge electrode allows more conductive hydrogel to be stored in the micropores, thereby slowing down the gel drying time, preventing signal degradation over time, and making such electrodes suitable for long-term recording. The gel in the micropores also acts as a buffer layer, helping to reduce movement between the electrode and the skin and reduce motion artifacts. Using such sponge electrodes, we demonstrated the recording of high-quality and robust ECG signals and electromyogram signals from skeletal muscle cells (biceps contraction) and smooth muscle cells (uterine contraction) (Figure 12b). This study shows that our sponge electrodes are a viable alternative to commercial Ag/AgCl electrodes or other flexible/stretchable thin-film electrodes for wearable ambulatory biopotential monitoring applications [106]. The engineering scheme of sponge-based porous conductive electrodes has a high practical reference value. The 3D interconnected porous PDMS skeleton loaded with conductive coatings stores trace electrolyte in micropores to slow gel drying and support continuous monitoring over 24 h. The porous structure disperses shear stress generated by movement and suppresses motion artifacts. The matched molding and spraying processes are simple for mass production, suitable for household ECG patches, and wearable sports monitoring devices.

### 4.2. Interface Biomimetics

Interface biomimetics focuses on the special wettability (super hydrophobicity, super amphiphilicity) and interface adhesion mechanisms of biological surfaces to improve the solid–liquid interface interaction between ECG electrodes and skin tissue and enhance biocompatibility and contact stability.

In long-term implantable ECG electrodes and wearable waterproof ECG device systems, the superhydrophobic–super electrolyte three-phase interface structure constructed by drawing on the lotus leaf effect in nature can significantly optimize the electrode working stability from the perspective of interface wettability regulation (Figure 12f). This biomimetic interface can effectively block the impact of sweat and body fluid leakage on electrode performance, and at the same time provide a continuous and unobstructed channel for the full contact between electrolytes and the skin, significantly improving the signal conduction efficiency and charge transfer characteristics at the interface, thereby effectively enhancing the long-term service stability and signal acquisition accuracy of implantable and wearable ECG electrodes, and providing a key interface design strategy for the practical application of long-term ECG monitoring [107]. In the field of implantable ECG electrodes and high-precision ECG sensors, biomimetic interface modification also shows great application potential. Functional modification of the electrode interface through biomimetic cell membrane structure surface modification or dopamine coating imitating mussel adhesion properties can reduce the immunogenicity and biotoxicity of electrode materials at the molecular level, effectively inhibit the inflammatory response and foreign body rejection produced by the body after implantation, and markedly boost the multifaceted performance of electrodes and contact stability of the electrode–skin/myocardial tissue interface. At the same time, such biomimetic interfaces are conducive to reducing interface impedance, enhancing the efficiency of ECG signal acquisition and transmission, and improving the accuracy of surface ECG signal recording and myocardial electrical signal monitoring, which have been widely used in cutting-edge biomedical fields such as clinical long-term ECG monitoring, implantable cardiac monitoring, and wearable ECG bracelets [29]. Weaken Wang et al. (Figure 12d) reported the process of constructing damage-resistant adhesive flexible skin-like ionic electrodes by creating hierarchical pores similar to Mytilus edulis through the recently developed solid-phase molecular self-assembly (SPMSA) strategy [108]. This strategy points out that for precipitates formed by a pair of oppositely charged polyelectrolytes and amphiphilic molecules, condensing the precipitate through slight mechanical pressure helps nanocluster amphiphilic molecules merge into large mesophases, thereby reducing interface energy and ultimately forming a macroscopically transparent supramolecular film. In this work, three polyelectrolytes were used instead of oppositely charged polyelectrolytes and amphiphilic molecules to introduce conductive ions and three-dimensional nanoscale channels into the film. Due to strong electrostatic interactions, oppositely charged polyelectrolyte chains cannot pair correctly before direct precipitation. Therefore, counterions are always sequestered in the precipitate to balance local osmotic pressure [109]. At the same time, hydrogen bonds between polyelectrolytes may promote further interactions between electrostatically interacting polyelectrolytes, thereby providing a nanoscale network in the film that balances the elastic and viscous properties of the film. Through this design, as long as there are still small connections in the damaged film, ion conduction remains constant, thereby providing stable real-time measurement signals even in the presence of cuts and fractures. In this way, we demonstrate for the first time the possibility of manufacturing damage-tolerant and robust flexible skin-like electrodes for human–computer interaction [52] (Figure 12e). Long-term, waterproof, and implantable ECG electrodes can be developed by learning from interface bionics technologies. The lotus leaf-inspired triphase super hydrophilic interface isolates the erosion of sweat and body fluid to stabilize electrode performance during long-term monitoring. The mussel dopamine multi-stage porous structure can be used to fabricate damage-resistant ECG electrodes; internal ion channels remain conductive even if the film cracks, so signals will not be interrupted under sweating and friction environments. The gel-free ionic biomimetic interface is friendly to sensitive skin and infants, avoiding gel allergy, and suitable for long-term household ECG screening and subcutaneous implantable ECG monitoring devices.

### 4.3. Mechanical Biomimetics

Mechanical biomimetics is committed to imitating the high elasticity, self-healing ability, and anisotropic mechanical response of biological tissues such as muscles, skin, and spider silk, to solve the problems of structural rupture and signal failure of ECG electrodes caused by skin deformation during human activities. In the field of flexible wearable ECG devices and dynamic ECG monitoring, the mechanical adaptability and structural stability of electrodes are key factors restricting their practical application. Stretchable ECG electrodes designed and prepared by drawing on the spring-like spiral structure and sarcomere sliding structure in organisms can maintain excellent electrical conductivity while endowing the electrode with excellent mechanical deformation tolerance. Such biomimetic structure electrodes can stably withstand complex deformations such as repeated bending, large-scale stretching, and multi-directional twisting during human activities, and still maintain the integrity of the conductive path and the stability of ECG signals during dynamic deformation, effectively solving the problems of traditional rigid ECG electrodes such as easy breakage, poor contact, and signal drift during human activities [110] (Figure 12g). With their excellent mechanical compliance, structural stability, and signal acquisition sensitivity, such biomimetic stretchable ECG electrodes show great potential in cutting-edge application scenarios such as flexible ECG patches, intelligent ECG clothing, and exercise ECG monitoring sensors, providing important structural design ideas and technical support for the development of the next generation of flexible, wearable, and highly adaptable ECG monitoring devices [111]. For example, Yangqin Ji et al. pointed out in their research that biomimetic artificial muscles (BMAMs) are artificial materials inspired by the structure and function of biological muscles, aiming to replicate the movement and function of real muscles [112]. Their excellent flexibility and stretchability enable these flexible materials to replace rigid transmission devices in specific fields of traditional mechanical systems [113]. The adoption of BMAM not only greatly simplifies the cumbersome and complex mechanical structure, but also simplifies the entire design process [113]. Therefore, in recent years, BMAM has attracted widespread attention in fields such as medical devices [114]. They have potential advantages in replacing rigid materials and applications in flexible transmission, making them a highly representative technological innovation in the current research field [115]. They successfully synthesized a series of composite electrode films doped with zinc oxide with different contents. The surface structure of the composite electrode films was comprehensively analyzed by scanning electron microscopy, and then their electrochemical behavior was evaluated in detail on an electrochemical workstation. Subsequently, we used a self-assembled BMAM output force test device to evaluate the force output of BMAMs produced by these composite electrode films. We conducted a comprehensive analysis to investigate the correlation between the electrochemical performance of the composite electrode films and the output force of BMAMs. The findings of these experiments deepen the understanding of the performance of composite electrode films and demonstrate their potential use in BMAMs [116].

### 4.4. Functional Biomimetics

Functional biomimetics breaks through the limitations of physical structure imitation, goes deep into the molecular mechanisms of biological perception, signal conduction, and energy conversion; endows ECG electrodes with environmental adaptability and bio-like intelligent response capabilities; and improves the intelligence level of ECG signal acquisition. In the fields of intelligent ECG monitoring and precise regulation of ECG signals, biomimetic functional ECG electrodes, relying on their unique electrochemical interface and ion transport characteristics, can accurately simulate the signal conduction mechanism of biological tissues by regulating ion migration and charge trapping behavior, realizing precise acquisition, filtering, and amplification of ECG signals (Figure 12g). Such biomimetic devices can efficiently complete the transmission, integration, and noise reduction in ECG signals, providing core support for the construction of high-precision, low-power intelligent ECG monitoring systems. In the research of biomimetic ECG sensors and intelligent diagnosis systems, by simulating the perception, conversion, and preprocessing mechanisms of electrical signals by human cardiomyocytes and nerve cells, biomimetic ECG sensors with high signal sensitivity, wide response range, and excellent mechanical flexibility can be designed and prepared [117]. Such devices can achieve efficient capture and preprocessing of weak ECG signals, simplify the subsequent signal analysis process, reduce system power consumption, and significantly improve the real-time performance and accuracy of ECG monitoring, laying an important material and structural foundation for the development of intelligent ECG diagnosis devices, remote ECG monitoring systems, and biomimetic ECG repair devices [34]. For example, Daniel Mihai Teeline et al. detailed the comprehensive development process of an artificial nerve simulator, including material selection, manufacturing process, structural and electrical characteristic analysis, and verification using experimental electrode assemblies. We demonstrated that the impedance value of the simulator at 1 kHz (~2 Ki) is highly matched with in vivo peripheral nerve measurements and supports stable, repeatable signal transmission. Finally, we discuss its potential applications not only as a pre-implantation test platform for implant modules, but also as a multifunctional tool for iterative electrode optimization, surgical training, and standardized benchmark testing of periprosthetic devices. By connecting material innovation with engineering design, this work lays a safer, faster, and more efficient foundation for the development of the next generation of neural interfaces [118].

### 4.5. Critical Assessment of Technology Readiness Level (TRL)

#### 4.5.1. Structurally Bionic Electrodes

Five bionic configurations, including skin microtexture, gecko seta, octopus sucker, cactus sweat-guiding, and sponge porous structures, can be fabricated into thin films in the lab. Sponge electrodes have completed short-term 1–3 h ECG tests on dozens of subjects, which can cut interfacial impedance and raise SNR steadily, satisfying TRL 5 small-scale human validation. Electrospinning and compression molding support pilot trial preparation. Main bottlenecks: Micro-nano structures show poor batch uniformity, and roll forming easily damages microarchitectures with over 40% impedance fluctuation among batches. Dense anti-slip microprotrusions block vapor channels and cause baseline drift after 7 h of continuous wear, while cactus designs only cope with mild perspiration. Such structures merely improve physical contact and fail to solve long-term conductive degradation of dehydrated hydrogels.

Application scope: Small-batch matches for consumer sports wristbands with a short monitoring duration only. It cannot be applied to 24 h Holter or clinical continuous monitoring. Further breakthroughs in multi-scale integrated molding are required to reach TRL 6.

#### 4.5.2. Differentiated TRL Evaluation of Interfacial Bionic Electrodes

##### Ion-Conductive and Mussel Dopamine Adhesive Interfaces

In vitro electrochemical tests on ion transport and dopamine porous adhesion have been completed, and ex vivo porcine tissue tests prove sustained ion conduction after electrode damage. Only lab specimens are prepared without human wearable tests, which merely meets TRL 4 lab component verification. Limitations: Dopamine coating and SPMSA take a long time and produce a massive amount of waste liquid, incompatible with continuous film production. Sweat electrolytes break interfacial hydrogen bonds; existing tests last merely several hours without 7-day long-term data. Only cell assays are used to screen skin irritation risks, lacking animal chronic toxicity and human patch tests, which greatly hinder clinical translation.

##### Thermosensitive Reversible Adhesive Interfaces

Electrodes based on thermosensitive bioadhesive polymers finish 7-day ECG recording and 10-cycle repeated wear tests on small subject groups. Its low-temperature peel-off, impedance reduction, and artifact suppression outperform commercial Ag/AgCl electrodes, and its molding fits regular flexible patch lines, reaching TRL 5. Limitations: Epidermal temperature differences across body parts lead to uneven adhesion. Thermoresponsive performance degrades after heating–cooling cycles with a reuse limit of 10 times. Delamination easily occurs between functional layers and porous substrates, requiring optimized composite molding.

#### 4.5.3. Gradated TRL Classification of Material Bionic Electrodes

##### PEDOT:PSS-Conductive Polymers (Semi-Commercial Pilot Production)

Glycerol, ionic liquid, and block copolymer modified PEDOT:PSS films so that they could adopt mature spin-coating and spray-coating processes. Sponge composite electrodes finished controlled dynamic tests on hundreds of volunteers with complete SNR and stretch stability data and have been produced in small batches for civilian motion sensors. It owns the highest TRL among all bionic schemes and satisfies TRL 6 pilot-scale application limits. Limitations: The maximum elongation after modification only hits 75%, and severe limb torsion triggers irreversible rupture of conductive networks. The material shows weak sweat resistance, and high-conductivity fillers slightly impair skin compatibility, requiring low-irritation modification for clinical use.

##### Carbon/Metal Nanocomposites

Lab samples filled with carbon nanotubes or silver nanowires exhibit ultrahigh conductivity (TRL 4). Carbon sponge electrodes pass small-scale human exercise tests and reach TRL 5. Key drawbacks include easy oxidation of metallic nanofillers, skin penetration risk of shedding nanoparticles, and the absence of medical-grade coating mass production, which restricts large-scale medical application.

##### Brown Algae PSTP Conductive Hydrogel

Only one-pot lab synthesis, molecular interaction, short adhesion, and in vitro cell tests are completed, without human wear or long-term aging experiments, merely meeting TRL 3 lab mechanism verification. Bottlenecks: Dehydration cannot be eliminated in mass production; ionic conductivity drops over 16% after 72 h of wear. Low mechanical strength causes cracks after hundreds of stretches, while inconsistent component ratios between batches damage repeatability, making pilot scaling impossible.

#### 4.5.4. Mechanical Bionic Electrodes

Complete patch prototypes including epidermis–dermis layered, serpentine interconnect, and gradient micropillar structures are available. ECG tests under limb bending and large stretching prove an obvious reduction in interfacial shear and motion artifacts, verified by short-term wear on dozens of subjects, reaching TRL 5. Limitations: Integrated molding of multi-layer dissimilar materials is complex, with frequent delamination between elastomer and hydrogel layers. Photolithography for micropillars costs 8–12 times more than planar electrodes. The design only optimizes skin mechanical matching, lacking long-term in vivo anti-fibrosis data for myocardial and implantable neural electrodes. High costs block its upgrade to TRL 7 full commercialization.

#### 4.5.5. Multi-Functional Integrated Bionic Electrodes

Lab-made integrated films combining neuromorphic conductive, layered anti-artifact, and breathable armor structures are only tested under in vitro sweat and stretch conditions, with limited ex vivo tissue signal data and no systematic long-term human trials, falling into TRL 3–4. Bottlenecks: Superimposed modules bring performance trade-offs; highly porous breathable architectures break conductive continuity. Fabrication contains 8–12 steps with yield lower than 60%. Multimodal sensing and self-powering designs remain theoretical, lacking systematic optimization on power consumption, signal crosstalk, and long-term stability, creating a wide gap to consumer and clinical products.

#### 4.5.6. Maturity Ranking of All Technical Routes

Single material modification (PEDOT:PSS) > single structural/single interfacial patches (TRL 5) > nanocomposites and thermosensitive interfaces (TRL 4–5) > ion-adhesive interfaces, multi-layer mechanical integration and multi-functional composite systems (TRL 3–4). Pure hydrogels and multi-module integrated designs have no near-term industrialization prospects.

#### 4.5.7. Analysis of Differentiated Industrialization Paths

At present, only sponge-structured bionic electrodes based on PEDOT:PSS can be translated into small consumer electronics at TRL 7. Thermosensitive interfaces and layered mechanical patches merely fit niche short-duration sports monitoring. Ion-adhesive and multi-functional integrated electrodes require 3–5 years of iteration in materials, manufacturing, and clinical trials to realize gradual pilot scaling.

### 4.6. Multidimensional Synergistic Optimization Strategies for Electrical Interfaces

Integrating biomimetic design frameworks spanning structural, material, and functional dimensions, a holistic interface optimization protocol is established across four orthogonal categories—material engineering, biomimetic structuring, electrical stimulation topology, and back-end readout circuitry—to systematically reduce interfacial impedance and homogenize in vivo current distribution.

(1) Biomimetic Material Optimization

**Figure 13:**
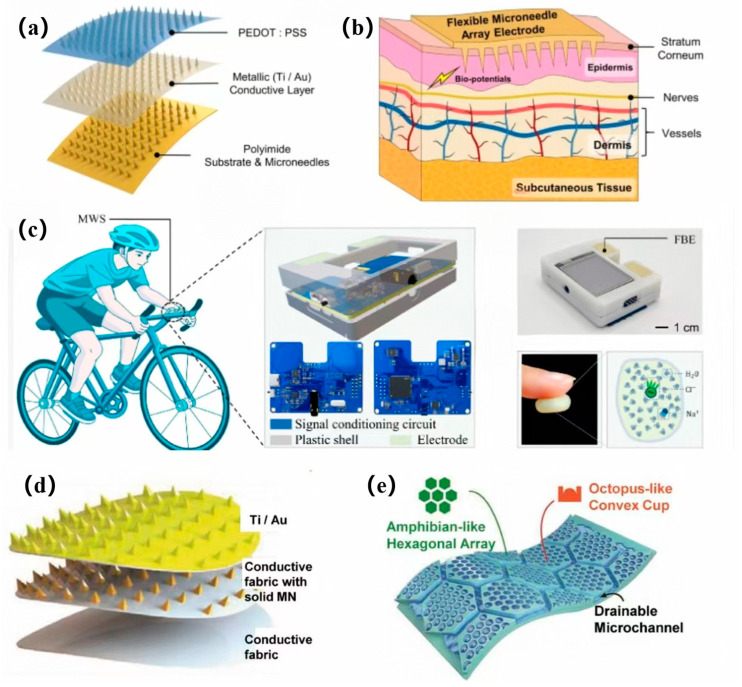
(**a**) Schematic view of the structure and material of the PI-MNA electrode [119]. (**b**) Principle of ultraminimally invasive biopotential acquisition by a flexible MNA electrode [119]. (**c**) Schematic illustrations of body-worn and handlebar-mounted MWS, its component breakdown, and photographs of fully assembled MWS are provided [120]. (**d**) Conductive fabric-supported microneedle patch for continuous ECG monitoring [120]. (**e**) Bioinspired adhesive patch combining octopus-like suction cups with tree-frog hexagonal toe-pad structures [121].

Alginate- and hyaluronic acid-based conductive hydrogels are deployed as interfacial coupling layers to replicate the high-water ionic microenvironment of native skin and establish continuous ionic transport pathways, lowering interfacial impedance by one to two orders of magnitude. PEDOT:PSS, a hybrid ion–electron conductive organic polymer, serves as the primary electrode material, facilitating simultaneous ionic and electronic charge transport to overcome the inherent limitation of conventional metallic electrodes that rely solely on surface charge exchange and substantially suppress EDL polarization. Silver nanowires and MXene nanofillers are incorporated into elastic substrates to construct neural-network-like interconnected conductive networks. Upon stretching or bending, conductive pathways autonomously slip and reorganize to avoid localized open circuits and resultant current discontinuities, sustaining stable in vivo current delivery (Figure 13a).

(2) Biomimetic Structural Optimization

Graded-modulus composite electrodes with stratified architecture are fabricated: a high-toughness elastic top layer bears tensile and shear mechanical stress, while a compliant ionic conductive underlayer conforms to microscale skin wrinkles. Contact area fluctuations between electrodes and skin are minimized during deformation, fundamentally inhibiting abrupt impedance shifts. Complementary biomimetic arrays featuring micropillars and multistage porous architectures offer dual benefits: dispersing interfacial current to eliminate localized polarization and generating breathable sweat drainage channels. This avoids continuous water film formation from accumulated sweat, which would otherwise trigger impedance drift and disrupt homogeneous in vivo current distribution (Figure 13b).

(3) Electrical Stimulation Topology Optimization

For wearable ECG arrays, MOP-inspired opposing electrode layouts are prioritized to balance superficial and deep-tissue signal capture requirements; MAP adjacent excitation is reserved for isolated localized superficial monitoring. A standard operating frequency of 50 kHz is adopted, consistent with optimal 3D electrical impedance tomography (3D-EIT) simulation parameters, supplemented by multi-frequency sweep detection at 10 kHz and 100 kHz. Low-frequency excitation strengthens ionic interfacial coupling, whereas high-frequency stimulation mitigates the high-impedance barrier of the stratum corneum to extend electric field penetration depth. A four-terminal hardware architecture with separated current injection and voltage readout channels completely isolates voltage measurements from interfacial impedance interference, enabling precise reconstruction of intrinsic current perturbations within biological tissues (Figure 13c).

(4) Back-End Electronic System Compensation Optimization

Aligned with the modular design paradigm of the readout circuit outlined above, a real-time interfacial impedance monitoring module is integrated into the microcontroller unit (MCU). The module continuously samples interfacial impedance across all channels and adaptively adjusts the output amplitude of direct digital synthesizer (DDS) excitation signals and the gain of instrumentation amplifiers to maintain constant excitation current injected into the human body. Center bandpass filters matched to the excitation frequency are embedded within signal pathways to filter low-frequency drift noise originating from interfacial polarization, collectively elevating the SNR of recorded ECG signals (Figure 13d,e).

## 5. Current Challenges and Development

### 5.1. Current Challenges Include a Lack of Design Coordination

Current biomimetic ECG electrodes struggle to achieve a harmonious integration of structure, function, and performance: (i) Structurally, the raised structures designed to mimic the micro- and nanotextures of the skin can block air channels, leading to skin discomfort during prolonged wear [122]. (ii) Mechanically, the insufficient strength of the flexible substrate causes curling and delamination, shortening the electrode’s lifespan; the root cause is the lack of a multidimensional, systematic optimization model for biomimetic systems. (iii) In complex scenarios, electrode performance deteriorates significantly: hydrophilic coatings tend to swell and peel off when exposed to sweat, leading to increased interfacial impedance and signal distortion; in extreme conditions, sweat corrosion and skin deformation exacerbate signal interference, potentially interrupting monitoring, whilst the electrodes lack sufficient resistance to dehydration. (iv) Large-scale production faces bottlenecks: mainstream photolithography and etching processes are costly and inefficient; the integrated fabrication of biomimetic structures and conductive layers remains immature, leading to delamination and increasing both costs and quality control difficulties. (v) Clinical validation is insufficient, largely confined to in vitro simulations or short-term human trials involving small sample sizes. There is a lack of large-scale, multi-center clinical, controlled trials, with very few patient-specific validations. Furthermore, inconsistent performance evaluation standards hinder clinical translation [123]. (vi) It is difficult to balance material properties: biomimetic materials exhibit poor conductivity, whilst the addition of conductive fillers compromises biocompatibility; furthermore, the mechanical properties, water resistance and skin adhesion of composite materials are mutually restrictive, remaining a core challenge in R&D.

### 5.2. Future Trends and Outlook

**Figure 14:**
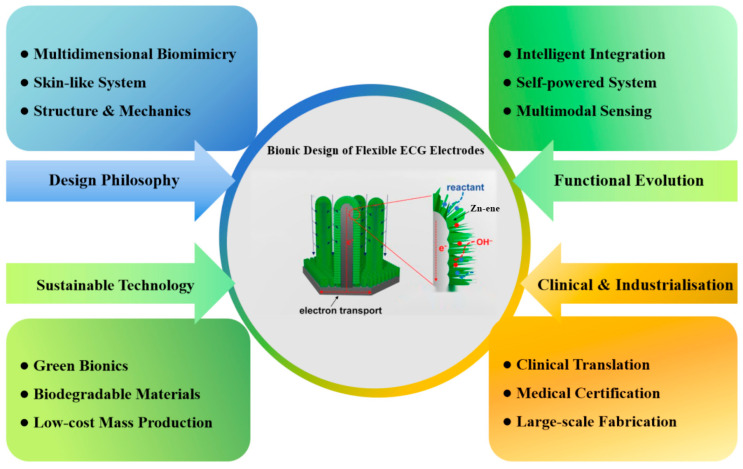
**Outlook of biomimetic ECG electrodes.** Schematic illustration of ion and CO2 diffusion, and electron transport in the leaf–branch–trunk electrode [129]. Copyright 2025 Carbon Energy.

In light of the current technical bottlenecks and clinical needs surrounding biomimetic ECG electrodes, future research will focus on the core principles of ‘system-level biomimicry, intelligent empowerment, environmental sustainability and clinical implementation’, to advance the technology from the laboratory stage towards industrialization and clinical application. In terms of design philosophy, we will shift from ‘single-dimensional biomimicry’ to multi-dimensional collaborative biomimicry encompassing ‘structure, interface and mechanics’, thereby constructing an integrated, skin-like electrode system. By mimicking the layered structure of the skin’s dermis, we have incorporated porous, breathable channels whilst ensuring a high degree of skin-conformity, thereby resolving the issue of discomfort caused by prolonged wear. Combined with mechanical simulations to optimize the modulus gradient of the substrate, the electrodes are capable of both adapting to the skin’s soft deformations and withstanding the mechanical stresses of daily stretching, thus achieving a comfortable and skin-friendly wearing experience alongside stable performance [124]. In terms of functional evolution, the next generation of biomimetic electrodes will undergo deep integration towards intelligent systems, overcoming the limitations of ‘passive monitoring’. The introduction of a dynamically cross-linked polymer substrate will enable self-repair of microcracks, thereby extending the electrode’s service life. Combined with piezoelectric or thermoelectric nanogenerators, the system will utilize body movement or temperature differentials to achieve self-powering, eliminating the need for batteries [125]. Furthermore, the integration of multi-modal sensing modules—such as temperature and pressure sensors—will enable the simultaneous monitoring of ECG and skin conditions, providing richer physiological data for clinical diagnosis [126]. In terms of technological sustainability, green bionic technology is set to become a key trend. To address the issue of electronic waste, the focus will be on developing conductive composites based on biodegradable bio-based materials such as chitosan and cellulose, enabling electrodes to degrade naturally once their monitoring tasks are complete, thereby reducing the environmental burden [127]. At the same time, efforts will be made to explore low-cost, large-scale manufacturing processes to transition bionic electrodes from being a ‘niche, high-end’ product to one that is ‘widely accessible for public use’, thereby truly integrating them into everyday health management scenarios [128]. In terms of clinical translation, the focus will be on developing high-signal-quality electrodes that meet clinical diagnostic standards. By optimizing interface impedance stability and resistance to motion artifacts, the electrodes will output signals that satisfy ECG clinical diagnostic requirements. Large-scale, multi-center clinical trials will be conducted to establish a unified performance evaluation system, thereby facilitating the acquisition of medical device certification for the electrodes. This will enable their widespread adoption in hospital wards and home care settings, providing a reliable tool for the early screening and remote diagnosis of conditions such as arrhythmia and myocardial ischemia. In terms of industrialization support, we will overcome the bottlenecks in the large-scale fabrication of micro- and nanoscale biomimetic structures, with a focus on developing low-cost, high-efficiency technologies such as 3D printing and electrospinning [125]. By utilizing the precise shaping capabilities of 3D printing to directly fabricate complex micro- and nanostructured substrates, and by mass-producing highly breathable fiber membrane electrodes via electrospinning, combined with roll-to-roll processes to achieve continuous production, we will significantly reduce manufacturing costs and lay a solid foundation for the industrialization of the technology (Figure 14).

## 6. Summary

This study systematically reviews the design philosophy, core technological approaches, and recent research progress of bio-inspired ECG electrodes. Adopting a biomimetic perspective and integrating interdisciplinary theories from biomedical engineering and materials science, it provides an in-depth analysis and identifies the key scientific bottlenecks—along with targeted solutions—regarding electrode adaptability at the biointerface, optimization of electrochemical performance, and enhancement of long-term wearing comfort. The research confirms that design strategies based on biomimetic principles can precisely align with the physiological characteristics of human skin and the transmission patterns of ECG signals. These strategies represent key technological pathways for overcoming the inherent limitations of traditional ECG electrodes in terms of adhesion stability, reliability of ECG signal acquisition, and long-term wearing comfort. Furthermore, it serves as the core driving force propelling wearable ECG monitoring technology from basic health screening towards clinical precision diagnosis, supplying systematic theoretical underpinnings and technical guidance for the clinical translation and industrial application of wearable medical devices.

Looking ahead, with continued breakthroughs in multi-dimensional collaborative bionic design (encompassing structural, material, and functional bionics), the integration of intelligent sensing capabilities, and eco-friendly manufacturing technologies, alongside interdisciplinary convergence, bionic ECG electrodes will further expand their applications across diverse fields such as wearable medical monitoring, remote health management, chronic disease prevention and control, and post-operative rehabilitation monitoring. This will provide a more robust technical foundation for the construction of an integrated ‘prevention–diagnosis–intervention’ healthcare management system, effectively facilitating the widespread adoption of professional medical monitoring technologies in the home setting. It will drive the evolution of healthcare management models from traditional, broad-brush approaches towards precision, convenience and personalization, thereby injecting new technological vitality into the development of the national health security system.

## Figures and Tables

**Table 1:** Quantitative comparison table of multi-dimensional performance of flexible ECG electrodes.

Electrode Type	Interface Impedance (kΩ@10 Hz)	Signal-to-Noise Ratio (dB)	Motion Artifact Suppression	Biocompatibility	Durability	Young’s Modulus (kPa)	Clinical Application Performance
Traditional Ag/AgCl Wet Electrode	<10, rises sharply after gel dries	30~40, <20 after exercise	Poor, baseline drift > 500 μV	Medium, prone to allergies with long-term wear	Poor, single-use, fails after >24 h	>1000, severe mismatch with skin modulus	Good, clinical gold standard; only suitable for static short-term monitoring
Common Flexible Dry Electrode	100~500, no gel ion pathway	15~25, severe thermal noise	Poor, baseline drift > 800 μV	Medium, poor air permeability, prone to skin inflammation	Medium, reusable, impedance drift > 50% after >72 h	100~500, insufficient flexibility	Poor, no clinical application cases; cannot meet diagnostic needs
Structural Bionic ECG Electrode	20~80, micro- and nanostructure increases contact area	25~35, anti-slip and noise reduction	Good, baseline drift < 150 μV	Good, porous structure improves air permeability	Good, reusable >10 times, stable for continuous wear > 7 days	50~200, reduced effective modulus	Medium, preliminary clinical verification; insufficient long-term stability
Interface Bionic ECG Electrode	10~30, human-like ion pathway, long-term drift < 10%	30~40, dynamic fluctuation < 5 dB	Excellent, baseline drift < 50 μV	Excellent, biocompatible material, no cytotoxicity	Excellent, reusable >20 times, no attenuation for continuous wear > 14 days	10~100, highly matched with skin	Good, small-sample clinical verification, meets diagnostic criteria; great commercialization potential
Material Bionic ECG Electrode	15~40, mixed ion–electron conductor	28~38, conductive network suppresses noise	Good, baseline drift < 200 μV	Excellent, natural polymer-based, low immunogenicity	Good, reusable >15 times, drift < 20% for continuous wear >10 days	5~50, excellent adaptability to soft tissues	Good, preclinical verification completed; no large-scale clinical data
Mechanical Bionic ECG Electrode	30~60, gradient modulus improves fitting	22~32, strain dispersion and noise reduction	Medium, baseline drift < 300 μV	Good, ultra-thin and low modulus, reduces mechanical irritation	Medium, reusable >5 times, stable for continuous wear > 5 days	1~30, tunable modulus, matches myocardium/skin	Medium, good preclinical verification for implantable scenarios; limited for transcutaneous monitoring
Functional Bionic ECG Electrode	<15, multi-dimensional optimization, close to wet electrode	35~45, high signal-to-noise ratio in both static and dynamic states	Excellent, baseline drift < 30 μV	Excellent, full-system biocompatible, no inflammatory reaction	Excellent, reusable >30 times, stable for continuous wear > 30 days	5~80, gradient distribution, optimal mechanical properties	Excellent, multi-center clinical control completed; suitable for full-scenario monitoring

**Table 2:** Definition criteria for each dimension.

Bionic Dimensions	Bionic Hierarchy	Objects of Biomimetic Optimization	References
Structural Bionics	Biogeometric Morphology	Skin fit, breathability and moisture-wicking, effective contact area	[24,25,26]
Interface Biomimicry	Molecular/ionic interactions at biointerfaces	Interface impedance, adhesion, sweat wicking	[27,28,29]
Bionic Materials	The chemical composition and molecular network structure of biological tissues.	Enhancing the intrinsic electrical conductivity, biocompatibility, and self-healing properties of materials	[30,31,32]
Mechanical Bionics	Gradients in the modulus of elasticity of biological tissues; stress-dissipating structures	Mechanical compatibility, tensile strength, slip resistance	[33]
Functional Bionics	The skin’s overall physiological functions	Integrated artifact suppression, long-lasting and stable sensing	[34]

**Table 3:** Comparison of various biomimetic approaches.

Representative Bionic Solutions	Advantages	Disadvantages	Suitable Scenarios	References
The structure of a gecko’s bristles	Increases the actual contact area between the electrode and the skin, significantly reducing interfacial impedance	The preparation process is complex, and the cost of template replication is high	Activities involving vigorous physical movement, such as running and working out	[37,38]
Mussel dopamine adhesion	Binds stably to the surface of sweaty or damp skin; highly biocompatible	Biological raw materials are unstable and prone to oxidation and degradation.	Continuous monitoring in the ward; post-operative ECG monitoring	[48,49]
Conductive hydrogel	High electrical conductivity	Hydrogels are prone to dehydration and drying out, leading to signal degradation during long-term monitoring	Disposable wearable health devices	[70,73]
Micropillar modulus gradient	Eliminates concentrated stress; causes minimal skin irritation	Mechanics—Difficulties with the conductive equilibrium	ECG monitoring at sites subject to significant movement (e.g., wrists, elbows)	[19,22,102]
Breathable skin electrodes	Resistance to signal drift; synergistic action of multiple mechanisms	Balancing the performance of multi-functional couplings	Multi-parameter integrated patient monitor	[13]

## Data Availability

No new data were created or analyzed in this study. Data sharing is not applicable.
